# The Effect of Semaglutide and GLP-1 RAs on Risk of Nonarteritic Anterior Ischemic Optic Neuropathy

**DOI:** 10.1016/j.ajo.2025.02.025

**Published:** 2025-02-25

**Authors:** NADIA J. ABBASS, RAYA NAHLAWI, JACQUELINE K. SHAIA, KEVIN C. ALLAN, DAVID C KAELBER, KATHERINE E. TALCOTT, RISHI P. SINGH

**Affiliations:** Case Western Reserve University School of Medicine (N.J.A., R.N., J.K.S.), Cleveland, Ohio, USA; Center for Ophthalmic Bioinformatics (N.J.A., R.N., J.K.S., K.E.T., R.P.S.), Cole Eye Institute, Cleveland Clinic, Cleveland, Ohio, USA; Cleveland Clinic Lerner College of Medicine of Case Western Reserve University (K.E.T., R.P.S.), Cleveland, Ohio, USA; Cleveland Clinic Cole Eye Institute (K.C.A., K.E.T., R.P.S.), Cleveland, Ohio, USA; Departments of Internal Medicine, Pediatrics and Population and Quantitative Health Sciences, Case Western Reserve University (D.C.K.), Cleveland, Ohio, USA; The Center for Clinical Informatics Research and Education (D.C.K.), The MetroHealth System, Cleveland, Ohio, USA; Cleveland Clinic Martin Hospitals (R.P.S.), Cleveland Clinic Florida, Stuart, Florida, USA

## Abstract

**PURPOSE:**

The association between GLP-1 receptor agonists (GLP-1RA) and nonarteritic anterior ischemic optic neuropathy (NAION) remains unclear. Given the de-bilitating sequelae of NAION and rapid increase of GLP-1RA use, further research is essential to investigate this potential relationship. This study seeks to determine the risk of NAION and ischemic optic neuropathy (ION) in patients prescribed GLP-1RAs.

**DESIGN:**

Retrospective matched cohort study.

**SETTING:**

TriNetX United States collaborative network.

**PARTICIPANTS:**

Patients ≥12 years old with type 2 diabetes (T2DM) and considered overweight or obese (high BMI), with at least one ophthalmology or neurology visit. Among T2DM patients, approximately 120,000 patients with a semaglutide prescription and 220,000 prescribed any GLP-1RA were compared to matched T2DM controls. Among high BMI patients, approximately 58,000 on semaglutide and 66,000 on any GLP-1RA were compared to matched controls.

**METHODS:**

Patients prescribed semaglutide or any GLP-1RA were compared with those on non-GLP-1RA medications. Populations were propensity matched (1:1) on various demographic and risk factors to balance base-line cohorts.

**MAIN OUTCOMES AND MEASURES:**

Cumulative incidence and risk of NAION and ION. Risk ratios (RR) with 95% confidence intervals (CI) were reported, with significance defined as CI <0.9 or > 1.1.

**RESULTS:**

In T2DM patients prescribed semaglutide, the risk of NAION (RR = 0.7, 95% CI: 0.523-0.937) and ION (RR = 0.788, 95% CI: 0.609-1.102) after 5 years was not significantly increased compared to matched T2DM controls. Similarly, T2DM patients on any GLP-1RA demonstrated no significant difference in the risk of NAION (RR = 0.887, 95% CI: 0.735-1.071) or ION (RR = 0.969, 95% CI: 0.813-1.154) compared to controls. Furthermore, no increased risk of either outcome was found in the high BMI groups prescribed semaglutide or any GLP-1RA. The cumulative 5-year risk of NAION and ION in T2DM patients on semaglutide was 0.065% and 0.08%, respectively. In those with high BMI prescribed semaglutide, the risk of NAION and ION after 2 years was 0.038% and 0.404%, respectively.

**CONCLUSIONS:**

There was no significant increase in risk of NAION or ION in patients taking semaglutide or GLP-1RAs compared to T2DM or high BMI controls.

Nonarteritic anterior ischemic optic neuropathy (NAION) is characterized by an acute, typically painless loss of vision that develops over hours to days.^1,2^ This condition affects an estimated 2.3 to 10.2 per 100,000 persons annually in the United States.^3,4^ Although the precise mechanism of optic disc ischemia remains unknown, NAION has been associated with various conditions, with hypothesized mechanisms including arteriosclerosis, vascular spasms, or adverse effects from medications.^5^ Identified systemic and cardiovascular risk factors for NAION include hypertension,^6–10^ hyperlipidemia,^10–12^ diabetes,^7–9, 13, 14^ and smoking. ^2,11,15^ Additional separate risk factors include hypotension,^16^ anemia,^17^ obstructive sleep apnea,^18^ and various coagulopathies.^19^ Furthermore, certain medications, such as phosphodiesterase-5 inhibitors^20,21^ and amiodarone,^22^ have also been linked to the development of NAION.

Glucagon like peptide-1 receptor agonists (GLP-1RA) are a class of medications that were originally approved for diabetes treatment with the notable side effect of pronounced weight loss.^23–25^ More recently, this class of medications has garnered numerous FDA approvals including weight loss and cardiovascular protection.^26^ This is thought to be due to potential risk factor mitigation, such as obesity, and anti-inflammatory properties.^27^ Within the ophthalmologic space, GLP-1RAs have most strongly been linked to a protective effect on glaucoma.^28–30^ However, one study demonstrated an increased risk of NAION in patients with type 2 diabetes mellitus (T2DM) or those who were overweight or obese (high BMI) taking semaglutide vs patients on non-GLP-1RAs antidiabetic medications. Additionally, the study found the risk of NAION was highest within the first year of starting semaglutide.^31^ However, the study included a relatively small cohort, with 46 NAION cases across all groups, and patients recruited from a single institution. A separate study evaluated this relationship among patients from 21 different countries and found no significant difference in the risk of NAION in high BMI and diabetic patients prescribed semaglutide. However, this study was not without limitations including overly broad ICD-10 coding, and restriction to white populations, which limits generalizability to the US due to known racial disparities in health in the US.^32^ While this study provides a valuable contribution to understanding this complex relationship, further research addressing these methodological shortcomings is warranted.

Our study builds on this work by investigating the association between semaglutide, NAION, and ischemic optic neuropathy (ION) among a large, diverse sample of US patients. We also expand upon past work by including ION (without exclusion criteria) as an additional outcome to capture a broader spectrum of potential associations. Additionally, we perform a secondary analysis of patients prescribed any GLP-1RA to evaluate whether the observed effects are specific to semaglutide or consistent across the entire class of medications.

## METHODS

This retrospective matched cohort study utilized the TriNetX US Collaborative Network, which is an electronic health records (EHRs) platform of approximately 116 million patients from 65 US healthcare institutions. All data in this platform is aggregated, deidentified, and searchable by International Classification of Diseases, Ninth and Tenth Revision (ICD9/10) codes. This retrospective study is exempt from informed consent. The data reviewed is a secondary analysis of existing data, does not involve intervention or interaction with human subjects, and is deidentified per the de-identification standard defined in Section §164.514(a) of the HIPAA Privacy Rule. The process by which the data is de-identified is attested to through a formal determination by a qualified expert as defined in Section §164.514(b)(1) of the HIPAA Privacy Rule. This formal determination by a qualified expert refreshed on December 2020.

To reflect previous studies investigating NAION association with GLP-1RA usage,^31^ several cohorts were designed as outlined in Table 1. We evaluated 2 patient cohorts; those with T2DM and those who were overweight or obese (high BMI). To improve diagnostic accuracy, all patients included in the study had Current Procedural Terminology (CPT) codes for ophthalmology services and procedures (1012793) or neurology and neuromuscular procedures (1013309), to ensure evaluation by ophthalmology and/or neurology specialists. The ICD-10, CPT, and RxNorm codes used are available in the supplementary material (E-Table 1). The CONSORT diagram (E-Figure 1.) illustrates the number of participants at each step of the inclusion and exclusion process.

Patients with T2DM were identified with ICD-10 code E11. The first cohort analyzed included T2DM patients with a semaglutide prescription meeting all criteria on or after December 5, 2017, the approval date by the Food and Drug Administration (FDA) for management of type 2 diabetes. The second cohort included T2DM patients on any GLP-1RA medication. This group included patients with prescriptions of semaglutide, liraglutide, lixisenatide, dulaglutide, tirzepatide, and exenatide, GLP-1RA medications approved for management of T2DM. For consistency in comparison, patients in the GLP-1RA group also met all inclusion and exclusion factors on or after December 5, 2017.

Patients with high BMI were identified as all patients with a BMI ≥ 25 kg/m^2^ at any point. For both the semaglutide and any GLP-1RA groups, patients were required to meet all inclusion and exclusion factors on or after June 4, 2021, the date semaglutide was FDA for weight loss in patients without diabetes. The GLP-1RA group included patients with high BMI with one or more prescriptions for semaglutide, liraglutide, or tirzepatide, the GLP-1RAs currently approved for weight loss. A sub-analysis was carried with the same cohorts, but including only patients ever having a BMI ≥ 30 kg/m^2^.

Two control cohorts were created, one for the T2DM analyses and one for the high BMI analyses. The T2DM control group included T2DM patients on non-GLP-1RA diabetes medications including sodium-glucose cotransporter-2 (SGLT2) inhibitors, dipeptidyl peptidase-4 (DPP-4) inhibitors, sulfonylureas, thiazolidinediones, metformin, insulin and insulin analogues, and alpha-glucosidase inhibitors. The high BMI control group included patients with a BMI ≥ 25 kg/m^2^ on non-GLP-1RA weight loss medications including setmelanotide, phentermine, naltrexone, orlistat, bupropion, and topiramate. Patients taking GLP-1RAs were excluded from both control cohorts.

The outcomes evaluated were ION and NAION. ION was identified using ICD-10 encounter diagnosis code H47.01. NAION cases were defined as patients with one or more ICD-10 encounter diagnosis code for ischemic optic neuropathy (H47.01), excluding those with codes for giant cell arteritis (GCA) (M31.5, M31.6), the leading cause of non-NAION cases of ischemic optic neuropathy. ION was chosen as a secondary outcome for 2 reasons (1) to confirm that excluding GCA reduced the number of ION cases and (2) to verify whether NAION and ION results were consistent, accounting for potential misclassification in the NAION analysis. These outcomes were evaluated at various time periods after the index event, with the index event representing the date the patient met all inclusion factors listed above. Outcomes in the T2DM cohort were evaluated at 1-, 3-, and 5-years, since semaglutide has been approved for the management of diabetes since 2017. For the overweight/obesity group, outcomes were evaluated at 1- and 2-years, after the approval of semaglutide for weight loss in 2021. Patients with the outcome prior to the index event were excluded.

To achieve balance amongst cohorts, 1:1 greedy propensity score matching (PSM) was performed using the TriNetX analytic feature (caliper = 0.25 SD) on patient demographics, NAION risk factors such as hypertension, indications and contraindications for semaglutide, factors related to high BMI, and medications associated with NAION including amiodarone and phosphodiesterase type 5 (PDE5) inhibitors. The full list of variables matched on can be found in Table 2. Information on race, ethnicity, and sex were drawn from the patient’s EHR and as such, may be self-reported or input by a staff member of the contributing HCO. A standard mean difference <0.1 was considered to be a successful covariate match. To further ensure the validity of the comparisons, a negative control (allergic contact dermatitis, ICD-10 L23) was evaluated as an outcome for each analysis as this condition is unlikely to be influenced by internal or systemic factors.

Cumulative incidence and risk ratios (RR) with 95% confidence intervals are reported. To avoid overinterpretation of small effects in a very large sample of patients, results with a CI <0.9 or >1.1 were considered to be statistically significant. All statistical analysis was carried out within the TriNetX platform. The Strengthening the Reporting of Observational Studies in Epidemiology (STROBE) guidelines were followed in the reporting of this study.

## RESULTS

### SEMAGLUTIDE VS MATCHED CONTROLS:

#### Type 2 diabetes mellitus

**FIGURE 1.**
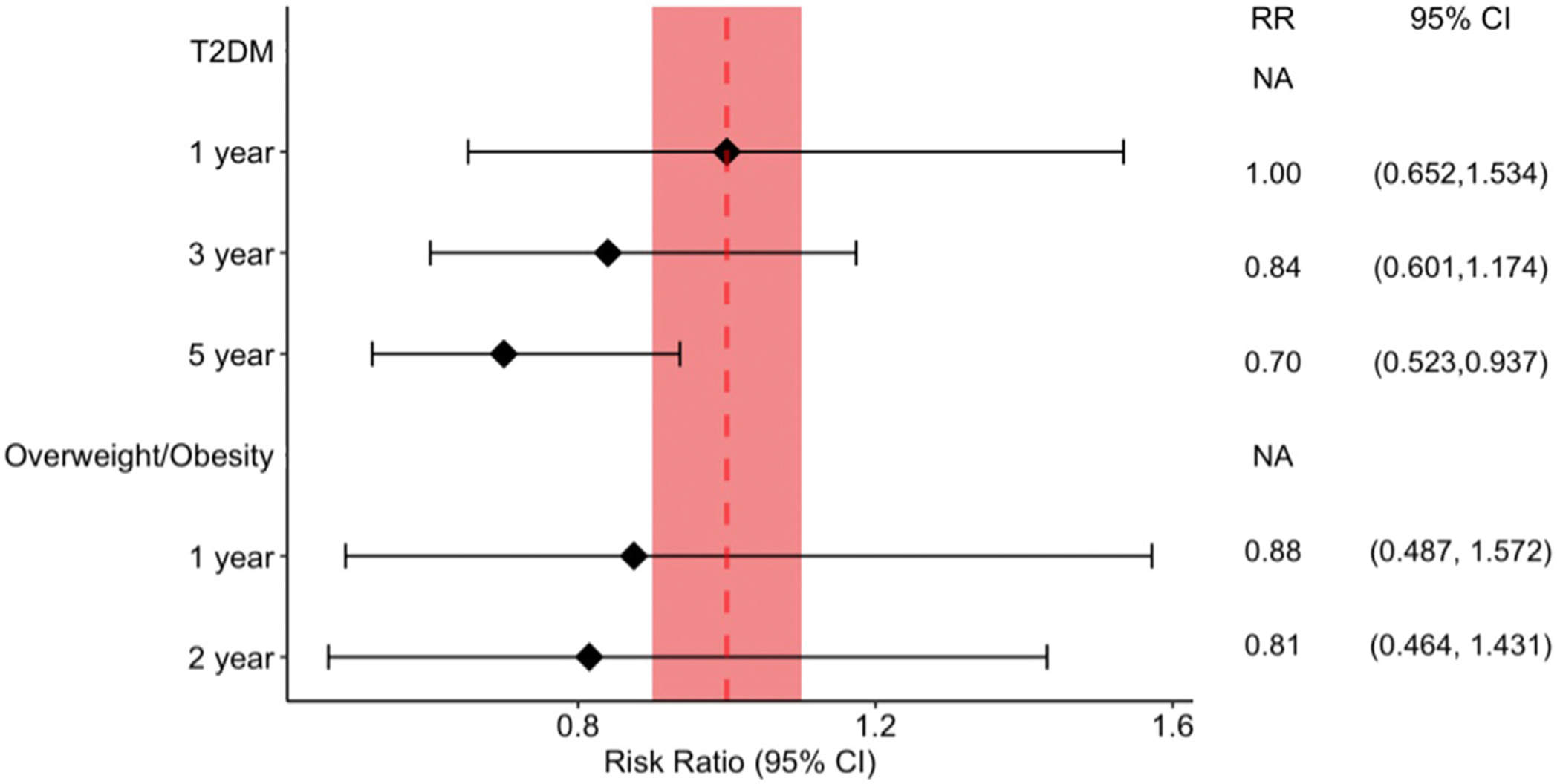
Risk ratio of NAION in patients prescribed semaglutide vs matched controls. *The shaded area represent the significance level used in this study.

T2DM patients prescribed semaglutide and non-GLP-1RA controls were propensity score matched, yielding approximately 130,000 patients in each cohort (Table 2, E-Tables 2–6), and compared for the risk of developing NAION. The semaglutide group had no increased risk of developing NAION at the 1 year- (RR 1.000, 95% CI 0.652-1.534), 3 year- (RR 0.840, 0.601-1.174), and 5-year time points (RR 0.700, 0.523-0.937) (Figure 1). The cumulative incidence of developing NAION in T2DM patients on semaglutide was 0.039%, 0.057%, and 0.065% at the 1, 3, and 5-year marks respectively. For ION, the relative risk was comparable between cases and controls at all timepoints ( *P* > .05). The cumulative incidence of 1-year, 3-year, and 5-year of developing ION in T2DM patients on semaglutide was 0.056%, 0.075%, and 0.080%, respectively.

#### High BMI

After PSM, approximately 58,000 patients with high BMI on semaglutide were compared to the same number of matched controls (E-Tables 7–10). The semaglutide group showed no significant difference in risk of developing NAION after 1 year (RR 0.875, 95% CI 0.487-1.572) or 2 years (RR 0.815, 0.464-1.431) compared to controls (Figure 1). The cumulative incidence of developing NAION in patients on semaglutide was 0.036% after 1 year and 0.038% after 2 years. Additionally, no difference in risk of ION was seen after 1 year (RR 0.769, 95% CI 0.4291.378) or 2 years (RR 0.735, 0.439-1.232). The cumulative 1- and 2-year incidence of developing ION in the semaglutide group was 0.035% and 0.043%, respectively.

### ALL GLP-1 RECEPTOR AGONISTS VS MATCHED CONTROLS:

#### Type 2 diabetes mellitus

**FIGURE 2.**
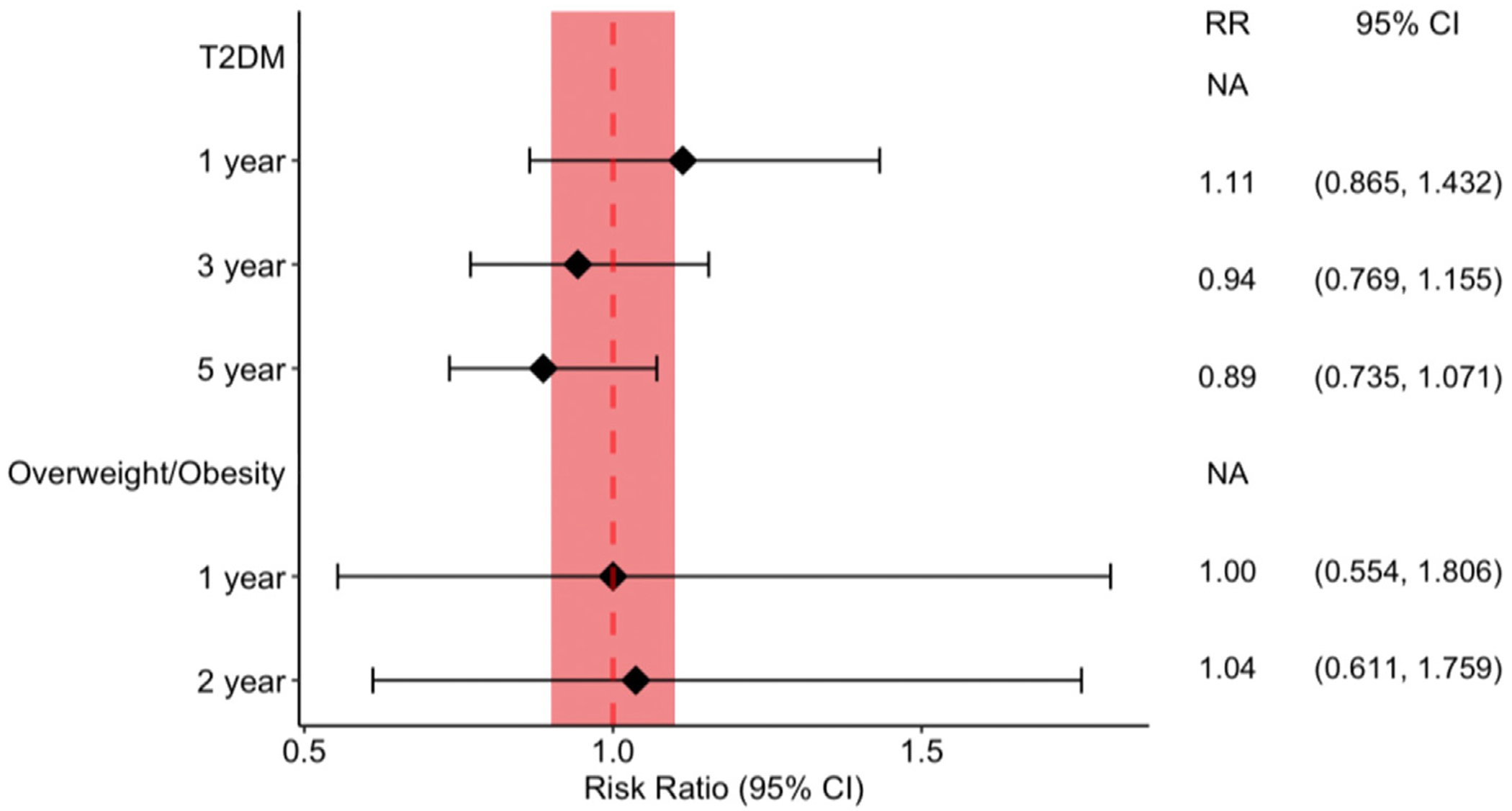
Risk ratio of NAION in patients prescribed any GLP-1RA vs matched controls. *The shaded area represent the significance level used in this study.

Approximately 222,000 T2DM patients prescribed any GLP-1RA approved for management of T2DM were compared to controls (E-Tables 11–16). There was no significant difference in the risk of NAION development after 1 (RR 1.113, 95% CI 0.865-1.432), 3 (RR 0.943, 0.769-1.155), or 5 years (RR 0.887, 0.735-1.071) between groups (Figure 2). The cumulative incidence of developing NAION in the GLP-1RA group was 0.059%, 0.081%, and 0.092% at 1, 3, and 5 years. Comparing the cohorts for risk of ION, there was no significant difference after 1 (RR 1.030, 95% CI 0.812-1.306), 3 (RR 0.981, 0.810-1.188), or 5 years (RR 0.969, 0.813-1.154). In the GLP-1RA group, the cumulative incidence of ION was 0.062% at 1 year, 0.093% at 3 years, and 0.107% at 5 years.

#### High BMI

The risk of NAION was evaluated in overweight or obese patients prescribed any GLP-1RA approved for weight loss management with PSM yielding approximately 66,000 study patients and matched controls (E-Tables 17–20). The risk of NAION after 1 year (RR 1.00, 95% CI 0.554-1.806) and 2 years (RR 1.037, 0.611-1.759) of medication prescription was comparable between cases and control patients (Figure 2).The cumulative incidence of NAION in the GLP-1RA group was 0.035% at 1 year and 0.043% at 2 years. There was also no difference in the risk of ION at 1 year (RR 1.091, 95% CI 0.612-1.945) with a cumulative incidence of 0.037%, and at 2 years (RR 1.297, 0.785-2.142) with a cumulative incidence of 0.056%.

##### BMI ≥ 30:

A sub-analysis of the high BMI cohort including only patients with obesity (BMI > 30) was performed. The risk of NAION for those prescribed semaglutide compared to non-GLP-1RAs was similar at 1- (RR 1.167, 0.6222.190) and 2- years (RR 1.080, 0.627-1.861). Additionally, there was no difference in risk of NAION in those prescribed any GLP-1RA compared to the same controls at 1- (RR 1.000, 0.561-1.782) or 2 years (RR 0.839, 0.498-1.412).

##### NEGATIVE CONTROL:

Importantly, for each comparison in this study, there were no significant differences for the risk of the negative control, allergic contact dermatitis, between cases and controls. (E-Table 21).

## DISCUSSION

This study sought to determine the risk of NAION in T2DM and obese or overweight patients prescribed semaglutide in a sample of over 116 million patients in the US healthcare network. Together, our findings demonstrate no difference in the risk of NAION or ION development among T2DM or high BMI patients prescribed semaglutide. When evaluating patients prescribed any GLP-1RA, the risk of NAION and ION were comparable between cases and controls.

Our findings differ from the results published by Hathaway et al, which demonstrated a significantly higher risk of NAION in T2DM and overweight/obesity patients on semaglutide. Their study also showed a 3-year cumulative incidence of 8.9% in the T2DM semaglutide group, significantly higher than the 0.057% reported in our study. There are several potential reasons for these differences. Patients in Hathaway et al’s study were recruited from a neuro-ophthalmology clinic at a tertiary care institute who very likely have significant underlying symptoms and neuro-ophthalmic diseases that distinctly differ from those of the general population taking GLP-1RAs, potentially causing a selection bias. Additionally, a strength of our analysis was the ability to evaluate NAION development at multiple time points. However, Hathaway et al’s study has a significant strength in that they were able to confirm a NAION diagnosis with clinical data.

Our conclusions align with those of Chou et al, who found no association between semaglutide and NAION. Although both studies utilized the same EHR platform, our study has several relative strengths. First, differences in cohort design allowed for larger cohorts in our study, resulting in substantially more outcome cases and narrower confidence intervals across all analyses. In Chou et al’s study, some comparisons reported ≤10 ION outcome cases due to de-identification which reports any number between 1 and 10 as 10, a feature of TriNetX’s de-identification standards, obscuring the exact numbers and introducing uncertainty to these comparisons. By creating larger cohorts, we were able to eliminate the rounding limitations that constrained several of their analyses. Another strength of our study is the requirement that all included patients were seen by neurology or ophthalmology. This is particularly important considering Chou et al’s reliance on the global TriNetX network, which includes patients from 21 countries likely with widely varying standards of care, access to ophthalmology services, prescribing practices, and semaglutide availability. Additionally, while Chou et al excluded patients with giant cell arteritis (GCA) in a sensitivity analysis, their main analysis did not apply this exclusion.^32^ In our study, exclusion of GCA resulted in a meaningful reduction of ischemic optic neuropathy cases. This approach is supported by findings in the study by Hathaway et al, who reported that 40% of cases coded as ischemic optic neuropathy were arteritic and associated with GCA upon manual review, further validating our exclusion methodology.^31^ Finally, by including patients of all races and utilizing data only in the US, our study enhances the generalizability of these findings to the US population.

From a pathophysiologic standpoint, GLP-1RAs target many of the systemic risk factors of NAION. Beyond their primary indication in treating T2DM and high BMI, these medications have demonstrated potential benefits in addressing many of the conditions associated with NAION including hypertension, hyperlipidemia, and obstructive sleep apnea.^33–35^ Although the exact pathophysiology of NAION is unknown, it is understood to result from insufficient blood flow to the optic nerve head. Potential causes of this ischemia include arteriosclerosis, thrombosis, embolism, hypoperfusion, and vasospasm.^5^ GLP-1RAs have been shown to impact multiple pathways involved in plaque formation, exhibiting atheroprotective effects.^36^ Importantly, since the pathophysiology of NAION is incompletely understood, there may be unaccounted factors that influence the relationship between these medications and this condition.

### LIMITATIONS:

This study has several limitations. First, with reliance on ICD coding, it is likely that some patients with ION and NAION were not captured and alternatively, some patients with the selected ICD-10 encounter diagnosis codes did not have a true diagnosis of ION or NAION. A previous study demonstrated that the ICD-10 code for ION has a 75% positive predictive value for NAION.^37^ To further refine the outcome of NAION, we utilized the ION ICD-10 code and excluded patients with ICD-10 codes for GCA. Although this reduced the number of cases, it likely did not exclude all non-NAION ION cases. To address this limitation, we also examined ION as an outcome, which re-inforced the NAION findings. Nonetheless, without access to the individual patient records, these diagnoses cannot be truly verified.

Furthermore, this study is limited by the inexistence of a neurology service code. As a proxy, we utilized the CPT code for Neurology and Neuromuscular Procedures. We additionally recognize that this CPT code does not capture the entire population of patients with a neurology visit. Another limitation studying these medications is the inherent difference between those prescribed GLP-1RAs, new and expensive drugs, compared to those who are not. One study demonstrated that of those medically eligible for this treatment, Asian, Black, Hispanic, and low-income patients are less likely to be treated with a GLP-1RAs.^38^ While we were able to match patients by race and ethnicity, we were unable to match on income and insurance status. Therefore, patients prescribed a GLP-1RA may be an overall healthier cohort, secondary to the impacts of the social determinants of health. Finally, we are not able to verify that the prescribed medications were dispensed or taken as directed or control for drug dosing.

## CONCLUSION

This study demonstrates no significant difference in the risk of NAION or ION in diabetic or overweight/obese patients prescribed semaglutide compared to matched controls on non-GLP-1RA medications at multiple time points. Furthermore, our secondary analysis, which included T2DM or high BMI patients on any GLP-1RA medication, supports the primary findings with no difference in the risk of either outcome compared to matched controls. These results support the more recent findings that demonstrate no difference in risk. The findings of these studies, along with the proven benefits of GLP-1RA medications and low overall prevalence of NAION should be considered together when counseling patients on the use of these medications.

## Supplementary Material

E-Figure 1

E-Table 1

E-Table 8

E-Table 4

E-Table 19

E-Table 21

E-Table 20

E-Table 14

E-Table 18

E-Table 17

E-Table 12

E-Table 16

E-Table 15

E-Table 13

E-Table 7

E-Table 10

E-Table 11

E-Table 9

E-Table 6

E-Table 5

E-Table 3

E-Table 2

Supplemental Material available at AJO.com.

## Figures and Tables

**TABLE 1.** Study Design, Inclusion, and Exclusion Criteria

	All Patients (+) Ophthalmology or Neurology Services [ >12 YO]			
	T2DM (+) T2DM [On/After Dec 5, 2017]		High BMI ( +) BMI > 25 [On/After June 4, 2021]	
	Study Group	Control Group	Study Group	Control Group
Semaglutide vs Non-GLP-1 RAs GLP-1 RAs vs Non-GLP-1RAs	(+) Semaglutide (+) All GLP-1 RA medications approved for T2DM^a^	(+) Insulin and analogues, Metformin, Sulfonylureas, α-glucosidase inhibitors, Thiazolidinediones, Dipeptidyl peptidase-4 inhibitors, Sodium-glucose transport protein 2 inhibitors (−) All GLP-1 RA Medications	(+) Semaglutide (+) All GLP-1 RA medications approved for weight loss ^b^	(+) Bupropion, Naltrexone, Orlistat, Topiramate, Phentermine, Setmelanotide (−)All GLP-1 RA

(+) = inclusion criteria, (−) = exclusion criteria, [] = filter.

a Tirzepatide, Liraglutide, Semaglutide, Lixisetanide, Dulaglutide, Exenatide.

b Tirzepatide, Liraglutide, Semaglutide.

**TABLE 2.** T2DM Cohort, Semaglutide vs Non-GLP-1RA Controls at 1 Year Before and After Propensity Score Matching (Nonarteritic Anterior Ischemic Optic Neuropathy^a^ )

Characteristic Name	Eligible Cohorts No. (%)			Cohorts After Matching No. (%)		
	Semaglutide ( *N* = 108400)	Non-GLP-1RA Diabetes Medications (*N* = 491407)	SMD	Semaglutide ( *N* = 107662)	Non-GLP-1RA Diabetes Medications ( *N* = 107662)	SMD
Current Age, Mean ( ± SD)	60.0 ± 12.6	67.0 (± 14.3)	0.525	60.2 ± 12.6	59.9 ± 13.7	0.025
Race						
*White*	61761 (57.00%)	275130 (56.00%)	0.02	61306 (56.90%)	61244 (56.90%)	0.001
*Black or African American*	24327 (22.40%)	107058 (21.80%)	0.016	24162 (22.40%)	24438 (22.70%)	0.006
*Hispanic or Latino*	10127 (9.30%)	54959 (11.20%)	0.061	10101 (9.40%)	9171 (8.50%)	0.03
Sex						
*Female*	61000 (56.30%)	233394 (47.50%)	0.176	60488 (56.20%)	61142 (56.80%)	0.012
BMI						
*BMI* (25-30 kg/*m2)*	30486 (28.10%)	180310 (36.70%)	0.184	30426 (28.30%)	31023 (28.80%)	0.012
*BMI* ( > 30 kg/*m2)*	77256 (71.30%)	242772 (49.40%)	0.459	76534 (71.10%)	76374 (70.90%)	0.003
Essential (primary) hypertension (I10)	88452 (81.60%)	366894 (74.70%)	0.168	87774 (81.50%)	87417 (81.20%)	0.009
Hyperlipidemia, unspecified (E78.5)	72897 (67.20%)	287805 (58.60%)	0.18	72275 (67.10%)	70632 (65.60%)	0.032
Sleep apnea (G47.3)	54256 (50.10%)	133109 (27.10%)	0.485	53534 (49.70%)	52724 (49.00%)	0.015
Other hyperlipidemia (E78.4)	34061 (31.40%)	125918 (25.60%)	0.129	33759 (31.40%)	32705 (30.40%)	0.021
Atherosclerotic heart disease of native coronary artery (I25.1)	24636 (22.70%)	128574 (26.20%)	0.08	24578 (22.80%)	24007 (22.30%)	0.013
Chronic kidney disease (CKD) (N18)	22122 (20.40%)	120280 (24.50%)	0.098	22062 (20.50%)	21861 (20.30%)	0.005
Acute pancreatitis (K85)	2184 (2.00%)	14339 (2.90%)	0.058	2183 (2.00%)	1777 (1.70%)	0.028
Malignant neoplasm of thyroid gland (C73)	1071 (1.00%)	3366 (0.70%)	0.033	1054 (1.00%)	838 (0.80%)	0.021
Other chronic pancreatitis (K86.1)	804 (0.70%)	8070 (1.60%)	0.083	804 (0.70%)	579 (0.50%)	0.026
Alcohol-induced chronic pancreatitis (K86.0)	55 (0.10%)	1384 (0.30%)	0.057	55 (0.10%)	54 (0.10%)	<0.001
Family history of multiple endocrine neoplasia [MEN] syndrome (Z83.41)	10 (0.00%)	22 (0.00%)	0.006	10 (0.00%)	10 (0.00%)	<0.001
Multiple endocrine neoplasia [MEN] type IIA (E31.22)	10 (0.00%)	34 (0.00%)	0.003	10 (0.00%)	10 (0.00%)	<0.001
Multiple endocrine neoplasia [MEN] type IIB (E31.23)	0 (0.00%)	10 (0.00%)	0.006	0 (0.00%)	10 (0.00%)	0.014
Sildenafil (136411)	10359 (9.60%)	32186 (6.50%)	0.111	10211 (9.50%)	9694 (9.00%)	0.017
Tadalafil (358263)	6400 (5.90%)	16967 (3.50%)	0.116	6271 (5.80%)	5652 (5.20%)	0.025
Amiodarone (703)	3298 (3.00%)	21595 (4.40%)	0.072	3294 (3.10%)	3062 (2.80%)	0.013
Vardenafil (306674)	1038 (1.00%)	4092 (0.80%)	0.013	1027 (1.00%)	842 (0.80%)	0.019
Avanafil (1291301)	162 (0.10%)	378 (0.10%)	0.022	158 (0.10%)	123 (0.10%)	0.009

a Different comparisons were ran for ION and NAION because giant cell arteritis was excluded in the cohort-building rather than the outcome stage.

## Abbreviations:

GLP-1: Glucagon-like peptide-1
ION: Ischemic Optic Neuropathy
NAION: Nonarteritic Ischemic Optic Neuropathy
T2DM: type 2 diabetes mellitus
RA: receptor agonists
EHRs: electronic health records
ICD9/10: International Classification of Diseases Ninth and Tenth Revision
HIPAA: Health Insurance Portability and Accountability Act
SGLT2: sodium-glucose cotransporter-2
DPP-4: dipeptidyl peptidase-4
FDA: Food and Drug Administration
PDE5: phosphodiesterase type 5
STROBE: Strengthening the Reporting of Observational Studies in Epidemiology
CPT: Current Procedural Terminology

## References

[1] Swartz NG, Beck RW, Savino PJ, Pain in anterior ischemic optic neuropathy. J Neuro-Ophthalmol Off J North Am Neuro-Ophthalmol Soc. 1995;15(1):9–10. doi:10.3109/01658109509044588. 7780575

[2] Characteristics of patients with nonarteritic anterior ischemic optic neuropathy eligible for the ischemic optic neuropathy decompression trial. Arch Ophthalmol. 1996;114(11):1366–1374. doi:10.1001/archopht.1996.01100140566007. 8906027

[3] Hattenhauer MG, Leavitt JA, Hodge DO, Grill R, Gray DT. Incidence of nonarteritic anterior ischemic optic neuropathy. Am J Ophthalmol. 1997;123(1):103–107. doi:10.1016/S0002-9394(14)70999-7. 9186104

[4] Johnson LN, Arnold AC. Incidence of nonarteritic and arteritic anterior ischemic optic neuropathy. Population-based study in the state of Missouri and Los Angeles County, California. J Neuro-Ophthalmol Off J North Am Neuro-Ophthalmol Soc. 1994;14(1):38–44. 8032479

[5] Miller NR, Arnold AC. Current concepts in the diagnosis, pathogenesis and management of nonarteritic anterior ischaemic optic neuropathy. Eye. 2015;29(1):65–79. doi:10.1038/eye.2014.144. 24993324 PMC4289822

[6] Cestari DM, Gaier ED, Bouzika P, Demographic, systemic, and ocular factors associated with nonarteritic anterior ischemic optic neuropathy. Ophthalmology. 2016;123(12):2446–2455. doi:10.1016/j.ophtha.2016.08.017. 27659545

[7] Guyer DR, Miller NR, Auer CL, Fine SL. The risk of cerebrovascular and cardiovascular dis ease in patients with anterior ischemic optic neuropathy. Arch Ophthalmol. 1985;103(8):1136–1142. doi:10.1001/archopht.1985.01050080048018. 4026642

[8] Hayreh SS, Joos KM, Podhajsky PA, Long CR. Systemic diseases associated with nonarteritic anterior ischemic optic neuropathy. Am J Ophthalmol. 1994;118(6):766–780. doi:10.1016/S0002-9394(14)72557-7. 7977604

[9] Repka MX, Savino PJ, Schatz NJ, Sergott RC. Clinical profile and long-term implications of anterior ischemic optic neuropathy. Am J Ophthalmol. 1983;96(4):478–483. doi:10.1016/S0002-9394(14)77911-5. 6624829

[10] Giambene B, Sodi A, Sofi F, Evaluation of traditional and emerging cardiovascular risk factors in patients with non-arteritic anterior ischemic optic neuropathy: a case-control study. Graefes Arch Clin Exp Ophthalmol . 2009;247(5):693–697. doi:10.1007/s00417-008-0981-6. 19052769

[11] Talks SJ, Chong NHV, Gibson JM, Dodson PM. Fibrinogen, cholesterol and smoking as risk factors for non-arteritic anterior ischaemic optic neuropathy. Eye. 1995;9(1):85–88. doi:10.1038/eye.1995.13. 7713255

[12] Salomon O, Huna-Baron R, Kurtz S, Analysis of prothrombotic and vascular risk factors in patients with nonarteritic anterior ischemic optic neuropathy. Ophthalmology. 1999;106(4):739–742. doi:10.1016/S0161-6420(99)90159-8. 10201595

[13] Jacobson DM, Vierkant RA, Belongia EA. Nonarteritic anterior ischemic optic neuropathy. A case-control study of potential risk factors. Arch Ophthalmol Chic Ill 1960. 1997;115(11):1403–1407. doi:10.1001/archopht.1997.01100160573008. 9366670

[14] Lee MS, Grossman D, Arnold AC, Sloan FA. Incidence of nonarteritic anterior ischemic optic neuropathy: increased risk among diabetic patients. Ophthalmology . 2011;118(5):959–963. doi:10.1016/j.ophtha.2011.01.054. 21439645 PMC3087834

[15] Chung SM, Gay CA, McCrary JA. Nonarteritic ischemic optic neuropathy. The impact of tobacco use. Ophthalmology. 1994;101(4):779–782. doi:10.1016/s0161-6420(94)31266-8. 8152775

[16] Hayreh S, Podhajsky P, Zimmerman MB. Role of nocturnal arterial hypotension in optic nerve head ischemic disorders. Ophthalmologica. 1999;213(2):76–96. doi:10.1159/000027399. 9885384

[17] Brouzas D, Charakidas A, Ladas I, Apostolopoulos M. Nonarteritic anterior ischemic optic neuropathy associated with chronic anemia: a case series of myelodysplastic syndrome patients. Clin Ophthalmol Auckl NZ. 2009;3:133–137. PMC2709018 19668557

[18] Bilgin G, Koban Y, Arnold AC. Nonarteritic anterior ischemic optic neuropathy and obstructive sleep apnea. J Neuroophthalmol. 2013;33(3):232. doi:10.1097/WNO.0b013e31828eecbd. 23719289

[19] Salomon O, Rosenberg N, Steinberg DM, Nonarteritic anterior ischemic optic neuropathy is associated with a specific platelet polymorphism located on the glycoprotein ib α gene. Ophthalmology. 2004;111(1):184–188. doi:10.1016/j.ophtha.2003.05.006. 14711733

[20] Pomeranz HD, Smith KH, Hart WM, Egan RA. Sildenafil-associated nonarteritic anterior ischemic optic neuropathy. Ophthalmology. 2002;109(3):584–587. doi:10.1016/S0161-6420(01)00976-9. 11874765

[21] Bollinger K, Lee MS. Recurrent visual field defect and ischemic optic neuropathy associated with Tadalafil rechallenge. Arch Ophthalmol. 2005;123(3):400–401. doi:10.1001/archopht.123.3.400. 15767488

[22] Macaluso DC, Shults WT, Fraunfelder FT. Features of amiodarone-induced optic neuropathy. Am J Ophthalmol. 1999;127(5):610–612. doi:10.1016/S0002-9394(99)00016-1. 10334361

[23] Shah M, Vella A. Effects of GLP-1 on appetite and weight. Rev Endocr Metab Disord. 2014;15(3):181–187. doi:10.1007/s11154-014-9289-5. 24811133 PMC4119845

[24] Pi-Sunyer X, Astrup A, Fujioka K, Randomized A, Controlled trial of 3.0 mg of Liraglutide in weight management. N Engl J Med. 2015;373(1):11–22. doi:10.1056/NEJMoa1411892. 26132939

[25] Wilding JPH, Batterham RL, Calanna S, Once-weekly semaglutide in adults with overweight or obesity. N Engl J Med. 2021;384(11):989–1002. doi:10.1056/NEJMoa2032183. 33567185

[26] FDA Approves First Treatment to Reduce Risk of Serious Heart Problems Specifically in Adults with Obesity or Overweight. FDA. 2024. Accessed December 11, 2024. https://www.fda.gov/news-events/press-announcements/fda-approves-first-treatment-reduce-risk-serious-heart-problems-specifically-adults-obesity-or

[27] Mehdi SF, Pusapati S, Anwar MS, Glucagon-like peptide-1: a multi-faceted anti-inflammatory agent. Front Immunol . 2023;14:1148209. doi:10.3389/fimmu.2023.1148209. 37266425 PMC10230051

[28] Niazi S, Gnesin F, Thein AS, Association between glucagon-like peptide-1 receptor agonists and the risk of glaucoma in individuals with type 2 diabetes. Ophthalmology. 2024;131(9):1056–1063. doi:10.1016/j.ophtha.2024.03.004. 38490274

[29] Sterling J, Hua P, Dunaief JL, Cui QN, VanderBeek BL. Glucagon-like peptide 1 receptor agonist use is associated with reduced risk for glaucoma. Br J Ophthalmol. 2023;107(2):215–220. doi:10.1136/bjophthalmol-2021-319232. 34413054 PMC8857286

[30] Muayad J, Loya A, Hussain ZS, Comparative effects of glucagon-like peptide 1 receptor agonists and Metformin on glaucoma risk in patients with type 2 diabetes. Ophthalmology. 2024;0(0). doi:10.1016/j.ophtha.2024.08.023. 39182626

[31] Hathaway JT, Shah MP, Hathaway DB, Risk of nonarteritic anterior ischemic optic neuropathy in patients prescribed semaglutide. JAMA Ophthalmol. 2024. doi:10.1001/jamaophthalmol.2024.2296. PMC11223051 38958939

[32] Chou CC, Pan SY, Sheen YJ, Association between semaglutide and non-arteritic anterior ischemic optic neuropathy: a multinational population-based real-world study. Ophthalmology. 2024. doi:10.1016/j.ophtha.2024.10.030. 39491755

[33] Ribeiro-Silva JC, Tavares CAM, Girardi ACC. The blood pressure lowering effects of glucagon-like peptide-1 receptor agonists: a mini-review of the potential mechanisms. Curr Opin Pharmacol. 2023;69:102355. doi:10.1016/j.coph.2023.102355. 36857807

[34] Premji R, Nylen ES, Naser N, Gandhi S, Burman KD, Sen S. Lipid profile changes associated with SGLT-2 inhibitors and GLP-1 agonists in diabetes and metabolic syndrome. Metab Syndr Relat Disord. 2022;20(6):321–328. doi:10.1089/met.2022.0004. 35452324

[35] Le KDR, Le K, Foo F. The impact of glucagon-like peptide 1 receptor agonists on obstructive sleep apnoea: a scoping review. Pharmacy. 2024;12(1):11. doi:10.3390/pharmacy12010011. 38251405 PMC10801460

[36] Lecis D, Prandi FR, Barone L, Beyond the cardiovascular effects of glucagon-like peptide-1 receptor agonists: body slimming and plaque stabilization. Are new statins born? Biomolecules. 2023;13(12):1695. doi:10.3390/biom13121695. 38136567 PMC10741698

[37] Hamedani AG, Kim DS, Chaitanuwong P, Gonzalez LA, Moss HE, DeLott LB. Validity of administrative coding for nonarteritic ischemic optic neuropathy. J Neuroophthalmol . 2022. doi:10.1097/WNO.0000000000002163. PMC11338734 38706093

[38] Eberly LA, Yang L, Essien UR, Racial, ethnic, and socioeconomic inequities in glucagon-like peptide-1 receptor agonist use among patients with diabetes in the US. JAMA Health Forum. 2021;2(12):e214182. doi:10.1001/jamahealthforum.2021.4182. 35977298 PMC8796881

