# Supplementary figures and images for: The Effect of Semaglutide and GLP-1 RAs on Risk of Nonarteritic Anterior Ischemic Optic Neuropathy
Source: Am J Ophthalmol. Author manuscript; Available in PMC 2026 Apr 25. (PMC13110070; doi:10.1016/j.ajo.2025.02.025)

**E-Figure 1.** CONSORT Diagram.

**
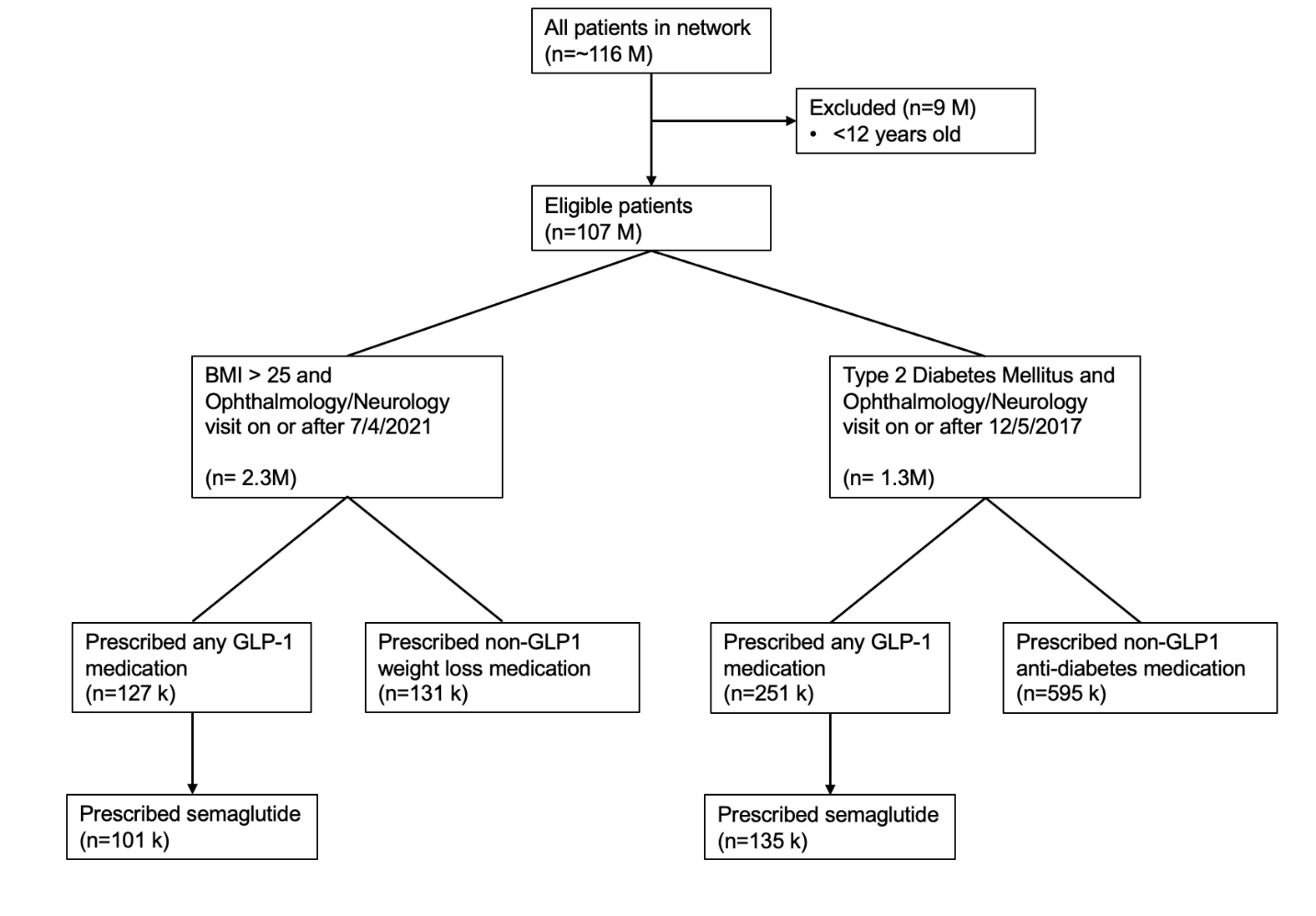
**

Supplement: E-Figure 1 [file NIHMS2163178-supplement-E-Figure_1.docx]
