# Supplementary material for: The Effect of Semaglutide and GLP-1 RAs on Risk of Nonarteritic Anterior Ischemic Optic Neuropathy
Source: Am J Ophthalmol. Author manuscript; Available in PMC 2026 Apr 25. (PMC13110070; doi:10.1016/j.ajo.2025.02.025)
Supplement: E-Table 1 [file NIHMS2163178-supplement-E-Table_1.docx]

**E-Table 1.** Codes Used in Study Design

| **Code (Type: Code)** | **Diagnosis/ Prescription** |
| --- | --- |
| ICD10: E11 | Type 2 Diabetes Mellitus |
| ICD10: H47.01 | Ischemic optic neuropathy |
| ICD10: M31.5 | Giant cell arteritis with polymyalgia rheumatica |
| ICD10: M31.6 | Other giant cell arteritis |
| ICD10: L23 | Allergic contact dermatitis |
| CPT: 1012793 | Ophthalmology Services and Procedures |
| CPT: 1013309 | Neurology and Neuromuscular Procedures |
| RXNORM:1991302 | Semaglutide |
| RXNORM:2601723 | Tirzepatide |
| RXNORM:475968 | Liraglutide |
| RXNORM:1440051 | Lixisetanide |
| RXNORM:1551291 | Dulaglutide |
| RXNORM:60548 | Exenatide |
| RXNORM:6809 | Metformin |
| ATC:A10A | Insulin and analogues |
| ATC:A10BB | Sulfonylureas |
| ATC:A10BF | α-glucosidase inhibitors |
| ATC:A10BG | Thiazolidinediones |
| ATC:A10BH | Dipeptidyl peptidase-4 inhibitors |
| ATC:A10BK | Sodium-glucose transport protein 2 inhibitors |
| RXNORM:42347 | Bupropion |
| RXNORM:7243 | Naltrexone |
| RXNORM:37925 | Orlistat |
| RXNORM:38404 | Topiramate |
| RXNORM:8152 | Phentermine |
| RXNORM:2469247 | Setmelanotide |
