# Supplementary material for: The Effect of Semaglutide and GLP-1 RAs on Risk of Nonarteritic Anterior Ischemic Optic Neuropathy
Source: Am J Ophthalmol. Author manuscript; Available in PMC 2026 Apr 25. (PMC13110070; doi:10.1016/j.ajo.2025.02.025)
Supplement: E-Table 4 [file NIHMS2163178-supplement-E-Table_4.docx]

**E-Table 4.** T2DM Cohort, Semaglutide vs. Non-GLP-1 RA Controls at 1 Year Before and After Propensity Score Matching (Ischemic Optic Neuropathy)

|  | **Eligible Cohorts** No. (%) | | | **Cohorts After Matching** No. (%) | | |
| --- | --- | --- | --- | --- | --- | --- |
| **Characteristic Name** | **semaglutide (N= 126979)** | **Non-GLP-1 RA Diabetes Medications (N= 563742)** | **SMD** | **semaglutide (N= 126129)** | **Non-GLP-1 RA Diabetes Medications (N= 126129)** | **SMD** |
| Current Age, Mean (+/- SD) | 60.09 (+/-12.66) | 67.01 (+/- 14.28) | 0.512 | 60.21 (+/- 12.59) | 59.88 (+/- 13.71) | 0.026 |
| Race |  |  |  |  |  |  |
| *White* | 76462 (60.22%) | 328241 (58.23%) | 0.041 | 75920 (60.19%) | 76119 (60.35%) | 0.003 |
| *Black or African American* | 25652 (20.20%) | 114352 (20.28%) | 0.002 | 25499 (20.22%) | 25645 (20.33%) | 0.003 |
| *Hispanic or Latino* | 12933 (10.19%) | 69312 (12.30%) | 0.067 | 12886 (10.22%) | 12060 (9.56%) | 0.022 |
| Sex |  |  |  |  |  |  |
| *Female* | 71880 (56.61%) | 269195 (47.75%) | 0.178 | 71286 (56.52%) | 71886 (56.99%) | 0.010 |
| BMI |  |  |  |  |  |  |
| *BMI (25-30 kg/m2)* | 34198 (26.93%) | 202087 (35.85%) | 0.193 | 34145 (27.07%) | 34685 (27.50%) | 0.010 |
| *BMI (>30 kg/m2)* | 85977 (67.71%) | 269391 (47.79%) | 0.412 | 85161 (67.52%) | 85100 (67.47%) | 0.001 |
| Essential (primary) hypertension (I10) | 104500 (82.30%) | 422913 (75.02%) | 0.178 | 103725 (82.24%) | 103363 (81.95%) | 0.007 |
| Hyperlipidemia, unspecified (E78.5) | 86887 (68.43%) | 332810 (59.04%) | 0.196 | 86153 (68.31%) | 84744 (67.19%) | 0.024 |
| Sleep apnea (G47.3) | 64717 (50.97%) | 156219 (27.71%) | 0.490 | 63892 (50.66%) | 62826 (49.81%) | 0.017 |
| Other hyperlipidemia (E78.4) | 35740 (28.15%) | 129585 (22.99%) | 0.118 | 35451 (28.11%) | 34171 (27.09%) | 0.023 |
| Atherosclerotic heart disease of native coronary artery (I25.1) | 31022 (24.43%) | 154434 (27.39%) | 0.068 | 30959 (24.55%) | 30303 (24.03%) | 0.012 |
| Chronic kidney disease (CKD) (N18) | 26484 (20.86%) | 141952 (25.18%) | 0.103 | 26425 (20.95%) | 26380 (20.92%) | 0.001 |
| Acute pancreatitis (K85) | 2678 (2.11%) | 16843 (2.99%) | 0.056 | 2674 (2.12%) | 2221 (1.76%) | 0.026 |
| Malignant neoplasm of thyroid gland (C73) | 1303 (1.03%) | 3907 (0.69%) | 0.036 | 1293 (1.03%) | 1093 (0.87%) | 0.016 |
| Other chronic pancreatitis (K86.1) | 951 (0.75%) | 9106 (1.62%) | 0.080 | 951 (0.75%) | 712 (0.57%) | 0.023 |
| Alcohol-induced chronic pancreatitis (K86.0) | 56 (0.04%) | 1492 (0.27%) | 0.056 | 56 (0.04%) | 62 (0.05%) | 0.002 |
| Family history of multiple endocrine neoplasia [MEN] syndrome (Z83.41) | 10 (0.01%) | 27 (0.01%) | 0.004 | 10 (0.01%) | 10 (0.01%) | 0.000 |
| Multiple endocrine neoplasia [MEN] type IIA (E31.22) | 10 (0.01%) | 42 (0.01%) | 0.000 | 10 (0.01%) | 11 (0.01%) | 0.001 |
| Multiple endocrine neoplasia [MEN] type IIB (E31.23) | 0 (0.00%) | 10 (0.00%) | 0.006 | 0 (0.00%) | 10 (0.01%) | 0.013 |
| Sildenafil (136411) | 11552 (9.10%) | 34583 (6.14%) | 0.112 | 11383 (9.03%) | 10761 (8.53%) | 0.017 |
| Tadalafil (358263) | 7222 (5.69%) | 18129 (3.22%) | 0.120 | 7074 (5.61%) | 6289 (4.99%) | 0.028 |
| Amiodarone (703) | 4039 (3.18%) | 26789 (4.75%) | 0.081 | 4033 (3.20%) | 3715 (2.95%) | 0.015 |
| Vardenafil (306674) | 1159 (0.91%) | 4282 (0.76%) | 0.017 | 1152 (0.91%) | 887 (0.70%) | 0.023 |
| Avanafil (1291301) | 175 (0.14%) | 395 (0.07%) | 0.021 | 172 (0.14%) | 128 (0.10%) | 0.010 |
