# Supplementary material for: The Effect of Semaglutide and GLP-1 RAs on Risk of Nonarteritic Anterior Ischemic Optic Neuropathy
Source: Am J Ophthalmol. Author manuscript; Available in PMC 2026 Apr 25. (PMC13110070; doi:10.1016/j.ajo.2025.02.025)
Supplement: E-Table 19 [file NIHMS2163178-supplement-E-Table_19.docx]

**E-Table 19.** High BMI Cohort, All GLP-1 RA vs. Non-GLP-1 RA Controls at 1 Year Before and After Propensity Score Matching (Ischemic Optic Neuropathy)

|  | **Eligible Cohorts** No. (%) | | | **Cohorts After Matching** No. (%) | | |
| --- | --- | --- | --- | --- | --- | --- |
| **Characteristic Name** | **All GLP-1 RA Medications**  **(N = 116,874)** | **Non-GLP-1 RA Diabetes Medications (N = 123,847)** | **SMD** | **All GLP-1 RA Medications**  **(N= 64,395)** | **Non-GLP-1 RA Diabetes Medications (N= 64,395)** | **SMD** |
| Current Age, Mean (+/- SD) | 116874 (100.00%) | 123847 (100.00%) | 0.277 | 64395 (100.00%) | 64395 (100.00%) | 0.012 |
| Race |  |  |  |  |  |  |
| *White* | 72341 (61.90%) | 89452 (72.20%) | 0.221 | 42946 (66.70%) | 43078 (66.90%) | 0.004 |
| *Black or African American* | 22105 (18.90%) | 15354 (12.40%) | 0.18 | 10207 (15.90%) | 10058 (15.60%) | 0.006 |
| *Hispanic or Latino* | 12467 (10.70%) | 10229 (8.30%) | 0.082 | 6016 (9.30%) | 5836 (9.10%) | 0.01 |
| Sex |  |  |  |  |  |  |
| *Female* | 73075 (62.50%) | 82011 (66.20%) | 0.077 | 42384 (65.80%) | 43961 (68.30%) | 0.052 |
| BMI |  |  |  |  |  |  |
| *BMI (25-30 kg/m2)* | 43136 (36.90%) | 72400 (58.50%) | 0.442 | 28180 (43.80%) | 27391 (42.50%) | 0.025 |
| *BMI (>30 kg/m2)* | 107460 (91.90%) | 89556 (72.30%) | 0.53 | 56587 (87.90%) | 57863 (89.90%) | 0.063 |
| Essential (primary) hypertension (I10) | 84175 (72.00%) | 60586 (48.90%) | 0.486 | 39329 (61.10%) | 39221 (60.90%) | 0.003 |
| Hyperlipidemia, unspecified (E78.5) | 69248 (59.30%) | 45325 (36.60%) | 0.466 | 30653 (47.60%) | 30307 (47.10%) | 0.011 |
| Sleep apnea (G47.3) | 60836 (52.10%) | 46794 (37.80%) | 0.29 | 30953 (48.10%) | 32353 (50.20%) | 0.043 |
| Other hyperlipidemia (E78.4) | 32544 (27.80%) | 19291 (15.60%) | 0.301 | 13454 (20.90%) | 13194 (20.50%) | 0.01 |
| Atherosclerotic heart disease of native coronary artery (I25.1) | 21800 (18.70%) | 14554 (11.80%) | 0.193 | 9809 (15.20%) | 9287 (14.40%) | 0.023 |
| Chronic kidney disease (CKD) (N18) | 19461 (16.70%) | 12606 (10.20%) | 0.191 | 8443 (13.10%) | 7954 (12.40%) | 0.023 |
| Acute pancreatitis (K85) | 2164 (1.90%) | 2804 (2.30%) | 0.029 | 1274 (2.00%) | 1235 (1.90%) | 0.004 |
| Malignant neoplasm of thyroid gland (C73) | 1149 (1.00%) | 1022 (0.80%) | 0.017 | 617 (1.00%) | 594 (0.90%) | 0.004 |
| Other chronic pancreatitis (K86.1) | 802 (0.70%) | 1319 (1.10%) | 0.041 | 555 (0.90%) | 561 (0.90%) | 0.001 |
| Alcohol-induced chronic pancreatitis (K86.0) | 42 (0.00%) | 280 (0.20%) | 0.053 | 42 (0.10%) | 34 (0.10%) | 0.005 |
| Family history of multiple endocrine neoplasia [MEN] syndrome (Z83.41) | 10 (0.00%) | 14 (0.00%) | 0.003 | 10 (0.00%) | 10 (0.00%) | <0.001 |
| Multiple endocrine neoplasia [MEN] type IIA (E31.22) | 11 (0.00%) | 18 (0.00%) | 0.005 | 10 (0.00%) | 10 (0.00%) | <0.001 |
| Multiple endocrine neoplasia [MEN] type IIB (E31.23) | 0 (0.00%) | 10 (0.00%) | 0.013 | 0 (0.00%) | 10 (0.00%) | 0.018 |
| Type 2 Diabetes Mellitus [T2DM] (E11) | 75578 (64.70%) | 23222 (18.80%) | 1.052 | 24048 (37.30%) | 22849 (35.50%) | 0.039 |
| Sildenafil (136411) | 9348 (8.00%) | 6754 (5.50%) | 0.102 | 4094 (6.40%) | 3783 (5.90%) | 0.02 |
| Tadalafil (358263) | 6109 (5.20%) | 4242 (3.40%) | 0.089 | 2603 (4.00%) | 2419 (3.80%) | 0.015 |
| Amiodarone (703) | 2796 (2.40%) | 2239 (1.80%) | 0.041 | 1402 (2.20%) | 1257 (2.00%) | 0.016 |
| Vardenafil (306674) | 919 (0.80%) | 565 (0.50%) | 0.042 | 358 (0.60%) | 344 (0.50%) | 0.003 |
| Avanafil (1291301) | 137 (0.10%) | 80 (0.10%) | 0.017 | 52 (0.10%) | 47 (0.10%) | 0.003 |
