# Supplementary material for: The Effect of Semaglutide and GLP-1 RAs on Risk of Nonarteritic Anterior Ischemic Optic Neuropathy
Source: Am J Ophthalmol. Author manuscript; Available in PMC 2026 Apr 25. (PMC13110070; doi:10.1016/j.ajo.2025.02.025)
Supplement: E-Table 21 [file NIHMS2163178-supplement-E-Table_21.docx]

**E-Table 21.** Risk ratio of Allergic Dermatitis (negative control) in study vs. control groups.

|  | Semaglutide vs. Controls | | All GLP-1 RAs vs. Controls | |
| --- | --- | --- | --- | --- |
|  | Risk Ratio | 95% Confidence Interval | Risk Ratio | 95% Confidence Interval |
| T2DM | | | | |
| 1 year | 1.102 | (0.947, 1.284) | 1.157 | (1.032, 1.297) |
| 3 years | 0.937 | (0.842,1.042) | 1.131 | (1.042, 1.227) |
| 5 years | 0.841 | (0.762, 0.928) | 1.059 | (0.984, 1.139) |
| Overweight/Obesity | | | | |
| 1 year | 0.958 | (0.777, 1.180) | 0.982 | (0.805, 1.199) |
| 2 years | 0.879 | (0.736, 1.050) | 0.955 | (0.802, 1.137) |
