# Supplementary material for: The Effect of Semaglutide and GLP-1 RAs on Risk of Nonarteritic Anterior Ischemic Optic Neuropathy
Source: Am J Ophthalmol. Author manuscript; Available in PMC 2026 Apr 25. (PMC13110070; doi:10.1016/j.ajo.2025.02.025)
Supplement: E-Table 20 [file NIHMS2163178-supplement-E-Table_20.docx]

**E-Table 20.** High BMI Cohort, All GLP-1 RA vs. Non-GLP-1 RA Controls at 2 Years Before and After Propensity Score Matching (Ischemic Optic Neuropathy)

|  | **Eligible Cohorts** No. (%) | | | **Cohorts After Matching** No. (%) | | |
| --- | --- | --- | --- | --- | --- | --- |
| **Characteristic Name** | **All GLP-1 RA Medications**  **(N = 114,272)** | **Non-GLP-1 RA Diabetes Medications (N = 119,099)** | **SMD** | **All GLP-1 RA Medications**  **(N= 62,621)** | **Non-GLP-1 RA Diabetes Medications (N= 62,621)** | **SMD** |
| Current Age, Mean (+/- SD) | 114272 (100.00%) | 119099 (100.00%) | 0.268 | 62621 (100.00%) | 62621 (100.00%) | 0.023 |
| Race |  |  |  |  |  |  |
| *White* | 68527 (60.00%) | 84056 (70.60%) | 0.224 | 40591 (64.80%) | 40899 (65.30%) | 0.01 |
| *Black or African American* | 23901 (20.90%) | 16402 (13.80%) | 0.19 | 10997 (17.60%) | 10689 (17.10%) | 0.013 |
| *Hispanic or Latino* | 12519 (11.00%) | 10321 (8.70%) | 0.077 | 6155 (9.80%) | 5955 (9.50%) | 0.011 |
| Sex |  |  |  |  |  |  |
| *Female* | 71716 (62.80%) | 78682 (66.10%) | 0.069 | 41370 (66.10%) | 42654 (68.10%) | 0.044 |
| BMI |  |  |  |  |  |  |
| *BMI (25-30 kg/m2)* | 42321 (37.00%) | 69336 (58.20%) | 0.434 | 27463 (43.90%) | 26804 (42.80%) | 0.021 |
| *BMI (>30 kg/m2)* | 104972 (91.90%) | 86332 (72.50%) | 0.523 | 55032 (87.90%) | 56203 (89.80%) | 0.059 |
| Essential (primary) hypertension (I10) | 82435 (72.10%) | 58751 (49.30%) | 0.48 | 38280 (61.10%) | 38197 (61.00%) | 0.003 |
| Hyperlipidemia, unspecified (E78.5) | 67605 (59.20%) | 44001 (36.90%) | 0.456 | 29609 (47.30%) | 29451 (47.00%) | 0.005 |
| Sleep apnea (G47.3) | 59195 (51.80%) | 45026 (37.80%) | 0.284 | 29954 (47.80%) | 31738 (50.70%) | 0.057 |
| Other hyperlipidemia (E78.4) | 32410 (28.40%) | 18877 (15.80%) | 0.305 | 13140 (21.00%) | 12950 (20.70%) | 0.007 |
| Atherosclerotic heart disease of native coronary artery (I25.1) | 21279 (18.60%) | 14165 (11.90%) | 0.188 | 9454 (15.10%) | 9116 (14.60%) | 0.015 |
| Chronic kidney disease (CKD) (N18) | 19043 (16.70%) | 12233 (10.30%) | 0.188 | 8180 (13.10%) | 7735 (12.40%) | 0.021 |
| Acute pancreatitis (K85) | 2122 (1.90%) | 2790 (2.30%) | 0.034 | 1254 (2.00%) | 1279 (2.00%) | 0.003 |
| Malignant neoplasm of thyroid gland (C73) | 1119 (1.00%) | 985 (0.80%) | 0.016 | 608 (1.00%) | 609 (1.00%) | <0.001 |
| Other chronic pancreatitis (K86.1) | 784 (0.70%) | 1309 (1.10%) | 0.044 | 559 (0.90%) | 560 (0.90%) | <0.001 |
| Alcohol-induced chronic pancreatitis (K86.0) | 42 (0.00%) | 288 (0.20%) | 0.055 | 41 (0.10%) | 40 (0.10%) | 0.001 |
| Family history of multiple endocrine neoplasia [MEN] syndrome (Z83.41) | 10 (0.00%) | 14 (0.00%) | 0.003 | 10 (0.00%) | 10 (0.00%) | <0.001 |
| Multiple endocrine neoplasia [MEN] type IIA (E31.22) | 10 (0.00%) | 10 (0.00%) | <0.001 | 10 (0.00%) | 10 (0.00%) | <0.001 |
| Multiple endocrine neoplasia [MEN] type IIB (E31.23) | 0 (0.00%) | 10 (0.00%) | 0.013 | 0 (0.00%) | 10 (0.00%) | 0.018 |
| Type 2 Diabetes Mellitus [T2DM] (E11) | 73947 (64.70%) | 22509 (18.90%) | 1.049 | 23318 (37.20%) | 22206 (35.50%) | 0.037 |
| Sildenafil (136411) | 9311 (8.10%) | 6634 (5.60%) | 0.102 | 1356 (2.20%) | 1240 (2.00%) | 0.013 |
| Tadalafil (358263) | 6025 (5.30%) | 4132 (3.50%) | 0.088 | 4021 (6.40%) | 3792 (6.10%) | 0.015 |
| Amiodarone (703) | 2731 (2.40%) | 2225 (1.90%) | 0.036 | 343 (0.50%) | 350 (0.60%) | 0.002 |
| Vardenafil (306674) | 915 (0.80%) | 550 (0.50%) | 0.043 | 2524 (4.00%) | 2506 (4.00%) | 0.001 |
| Avanafil (1291301) | 140 (0.10%) | 78 (0.10%) | 0.019 | 45 (0.10%) | 47 (0.10%) | 0.001 |
