# Supplementary material for: The Effect of Semaglutide and GLP-1 RAs on Risk of Nonarteritic Anterior Ischemic Optic Neuropathy
Source: Am J Ophthalmol. Author manuscript; Available in PMC 2026 Apr 25. (PMC13110070; doi:10.1016/j.ajo.2025.02.025)
Supplement: E-Table 18 [file NIHMS2163178-supplement-E-Table_18.docx]

**E-Table 18.** High BMI Cohort, All GLP-1 RA vs. Non-GLP-1 RA Controls at 2 Years Before and After Propensity Score Matching (Non-Arteritic Anterior Ischemic Optic Neuropathy)

|  | **Eligible Cohorts** No. (%) | | | **Cohorts After Matching** No. (%) | | |
| --- | --- | --- | --- | --- | --- | --- |
| **Characteristic Name** | **All GLP-1 RA Medications**  **(N = 120,314)** | **Non-GLP-1 RA Diabetes Medications (N = 126,435)** | **SMD** | **All GLP-1 RA Medications**  **(N= 66,030)** | **Non-GLP-1 RA Diabetes Medications (N= 66,030)** | **SMD** |
| Current Age, Mean (+/- SD) | 120314 (100.00%) | 126435 (100.00%) | 0.278 | 66030 (100.00%) | 66030 (100.00%) | 0.02 |
| Race |  |  |  |  |  |  |
| *White* | 72960 (60.60%) | 90126 (71.30%) | 0.226 | 43171 (65.40%) | 43525 (65.90%) | 0.011 |
| *Black or African American* | 24001 (19.90%) | 16469 (13.00%) | 0.187 | 11102 (16.80%) | 10901 (16.50%) | 0.008 |
| *Hispanic or Latino* | 13445 (11.20%) | 11034 (8.70%) | 0.082 | 6612 (10.00%) | 6311 (9.60%) | 0.015 |
| Sex |  |  |  |  |  |  |
| *Female* | 75505 (62.80%) | 83716 (66.20%) | 0.072 | 43692 (66.20%) | 45205 (68.50%) | 0.049 |
| BMI |  |  |  |  |  |  |
| *BMI (25-30 kg/m2)* | 44646 (37.10%) | 73944 (58.50%) | 0.438 | 29037 (44.00%) | 28218 (42.70%) | 0.025 |
| *BMI (>30 kg/m2)* | 110631 (92.00%) | 91473 (72.30%) | 0.53 | 58056 (87.90%) | 59343 (89.90%) | 0.062 |
| Essential (primary) hypertension (I10) | 86507 (71.90%) | 61631 (48.70%) | 0.487 | 40186 (60.90%) | 40036 (60.60%) | 0.005 |
| Hyperlipidemia, unspecified (E78.5) | 70665 (58.70%) | 45787 (36.20%) | 0.463 | 31011 (47.00%) | 30722 (46.50%) | 0.009 |
| Sleep apnea (G47.3) | 62218 (51.70%) | 47481 (37.60%) | 0.288 | 31502 (47.70%) | 33132 (50.20%) | 0.049 |
| Other hyperlipidemia (E78.4) | 33976 (28.20%) | 19803 (15.70%) | 0.307 | 13899 (21.00%) | 13549 (20.50%) | 0.013 |
| Atherosclerotic heart disease of native coronary artery (I25.1) | 22139 (18.40%) | 14640 (11.60%) | 0.192 | 9953 (15.10%) | 9345 (14.20%) | 0.026 |
| Chronic kidney disease (CKD) (N18) | 20029 (16.60%) | 12720 (10.10%) | 0.195 | 8566 (13.00%) | 8119 (12.30%) | 0.02 |
| Acute pancreatitis (K85) | 2227 (1.90%) | 2887 (2.30%) | 0.03 | 1351 (2.00%) | 1265 (1.90%) | 0.009 |
| Malignant neoplasm of thyroid gland (C73) | 1174 (1.00%) | 1035 (0.80%) | 0.017 | 644 (1.00%) | 599 (0.90%) | 0.007 |
| Other chronic pancreatitis (K86.1) | 834 (0.70%) | 1367 (1.10%) | 0.041 | 562 (0.90%) | 593 (0.90%) | 0.005 |
| Alcohol-induced chronic pancreatitis (K86.0) | 42 (0.00%) | 293 (0.20%) | 0.054 | 42 (0.10%) | 32 (0.00%) | 0.006 |
| Family history of multiple endocrine neoplasia [MEN] syndrome (Z83.41) | 10 (0.00%) | 14 (0.00%) | 0.003 | 10 (0.00%) | 10 (0.00%) | <0.001 |
| Multiple endocrine neoplasia [MEN] type IIA (E31.22) | 11 (0.00%) | 18 (0.00%) | 0.005 | 10 (0.00%) | 10 (0.00%) | <0.001 |
| Multiple endocrine neoplasia [MEN] type IIB (E31.23) | 0 (0.00%) | 10 (0.00%) | 0.013 | 0 (0.00%) | 10 (0.00%) | 0.017 |
| Type 2 Diabetes Mellitus [T2DM] (E11) | 77785 (64.70%) | 23682 (18.70%) | 1.052 | 24522 (37.10%) | 23309 (35.30%) | 0.038 |
| Sildenafil (136411) | 9670 (8.00%) | 6908 (5.50%) | 0.103 | 4138 (6.30%) | 3923 (5.90%) | 0.014 |
| Tadalafil (358263) | 6282 (5.20%) | 4317 (3.40%) | 0.089 | 2649 (4.00%) | 2513 (3.80%) | 0.011 |
| Amiodarone (703) | 2832 (2.40%) | 2263 (1.80%) | 0.04 | 1382 (2.10%) | 1247 (1.90%) | 0.015 |
| Vardenafil (306674) | 948 (0.80%) | 577 (0.50%) | 0.042 | 351 (0.50%) | 348 (0.50%) | 0.001 |
| Avanafil (1291301) | 143 (0.10%) | 81 (0.10%) | 0.018 | 50 (0.10%) | 43 (0.10%) | 0.004 |
