# Supplementary material for: The Effect of Semaglutide and GLP-1 RAs on Risk of Nonarteritic Anterior Ischemic Optic Neuropathy
Source: Am J Ophthalmol. Author manuscript; Available in PMC 2026 Apr 25. (PMC13110070; doi:10.1016/j.ajo.2025.02.025)
Supplement: E-Table 17 [file NIHMS2163178-supplement-E-Table_17.docx]

**E-Table 17.** High BMI Cohort, All GLP-1 RA vs. Non-GLP-1 RA Controls at 1 Year Before and After Propensity Score Matching (Non-Arteritic Anterior Ischemic Optic Neuropathy)

|  | **Eligible Cohorts** No. (%) | | | **Cohorts After Matching** No. (%) | | |
| --- | --- | --- | --- | --- | --- | --- |
| **Characteristic Name** | **All GLP-1 RA Medications**  **(N = 113,981)** | **Non-GLP-1 RA Diabetes Medications (N = 118,760)** | **SMD** | **All GLP-1 RA Medications**  **(N= 62,476)** | **Non-GLP-1 RA Diabetes Medications (N= 62,476)** | **SMD** |
| Current Age, Mean (+/- SD) | 113981 (100.00%) | 118760 (100.00%) | 0.269 | 62476 (100.00%) | 62476 (100.00%) | 0.022 |
| Race |  |  |  |  |  |  |
| *White* | 68347 (60.00%) | 83817 (70.60%) | 0.224 | 40442 (64.70%) | 40789 (65.30%) | 0.012 |
| *Black or African American* | 23833 (20.90%) | 16347 (13.80%) | 0.19 | 10982 (17.60%) | 10702 (17.10%) | 0.012 |
| *Hispanic or Latino* | 12499 (11.00%) | 10292 (8.70%) | 0.077 | 6178 (9.90%) | 5944 (9.50%) | 0.013 |
| Sex |  |  |  |  |  |  |
| *Female* | 71507 (62.70%) | 78424 (66.00%) | 0.069 | 41216 (66.00%) | 42522 (68.10%) | 0.044 |
| BMI |  |  |  |  |  |  |
| *BMI (25-30 kg/m2)* | 42193 (37.00%) | 69124 (58.20%) | 0.434 | 27411 (43.90%) | 26621 (42.60%) | 0.026 |
| *BMI (>30 kg/m2)* | 104708 (91.90%) | 86074 (72.50%) | 0.524 | 54867 (87.80%) | 56079 (89.80%) | 0.062 |
| Essential (primary) hypertension (I10) | 82176 (72.10%) | 58515 (49.30%) | 0.481 | 38205 (61.20%) | 38137 (61.00%) | 0.002 |
| Hyperlipidemia, unspecified (E78.5) | 67374 (59.10%) | 43809 (36.90%) | 0.456 | 29568 (47.30%) | 29405 (47.10%) | 0.005 |
| Sleep apnea (G47.3) | 59018 (51.80%) | 44863 (37.80%) | 0.284 | 29855 (47.80%) | 31593 (50.60%) | 0.056 |
| Other hyperlipidemia (E78.4) | 32287 (28.30%) | 18783 (15.80%) | 0.305 | 13173 (21.10%) | 12874 (20.60%) | 0.012 |
| Atherosclerotic heart disease of native coronary artery (I25.1) | 21164 (18.60%) | 14056 (11.80%) | 0.188 | 9441 (15.10%) | 9143 (14.60%) | 0.013 |
| Chronic kidney disease (CKD) (N18) | 18957 (16.60%) | 12148 (10.20%) | 0.189 | 8189 (13.10%) | 7788 (12.50%) | 0.019 |
| Acute pancreatitis (K85) | 2116 (1.90%) | 2781 (2.30%) | 0.034 | 1282 (2.10%) | 1292 (2.10%) | 0.001 |
| Malignant neoplasm of thyroid gland (C73) | 1116 (1.00%) | 978 (0.80%) | 0.016 | 616 (1.00%) | 606 (1.00%) | 0.002 |
| Other chronic pancreatitis (K86.1) | 781 (0.70%) | 1304 (1.10%) | 0.044 | 537 (0.90%) | 565 (0.90%) | 0.005 |
| Alcohol-induced chronic pancreatitis (K86.0) | 42 (0.00%) | 288 (0.20%) | 0.055 | 42 (0.10%) | 42 (0.10%) | <0.001 |
| Family history of multiple endocrine neoplasia [MEN] syndrome (Z83.41) | 10 (0.00%) | 14 (0.00%) | 0.003 | 10 (0.00%) | 10 (0.00%) | <0.001 |
| Multiple endocrine neoplasia [MEN] type IIA (E31.22) | 10 (0.00%) | 10 (0.00%) | <0.001 | 10 (0.00%) | 10 (0.00%) | <0.001 |
| Multiple endocrine neoplasia [MEN] type IIB (E31.23) | 0 (0.00%) | 10 (0.00%) | 0.013 | 0 (0.00%) | 10 (0.00%) | 0.018 |
| Type 2 Diabetes Mellitus [T2DM] (E11) | 73707 (64.70%) | 22386 (18.80%) | 1.049 | 23216 (37.20%) | 22087 (35.40%) | 0.038 |
| Sildenafil (136411) | 9293 (8.20%) | 6618 (5.60%) | 0.102 | 4012 (6.40%) | 3773 (6.00%) | 0.016 |
| Tadalafil (358263) | 6013 (5.30%) | 4122 (3.50%) | 0.088 | 2570 (4.10%) | 2477 (4.00%) | 0.008 |
| Amiodarone (703) | 2715 (2.40%) | 2207 (1.90%) | 0.036 | 1300 (2.10%) | 1269 (2.00%) | 0.003 |
| Vardenafil (306674) | 913 (0.80%) | 549 (0.50%) | 0.043 | 344 (0.60%) | 342 (0.50%) | <0.001 |
| Avanafil (1291301) | 140 (0.10%) | 77 (0.10%) | 0.019 | 49 (0.10%) | 45 (0.10%) | 0.002 |
