# Supplementary material for: The Effect of Semaglutide and GLP-1 RAs on Risk of Nonarteritic Anterior Ischemic Optic Neuropathy
Source: Am J Ophthalmol. Author manuscript; Available in PMC 2026 Apr 25. (PMC13110070; doi:10.1016/j.ajo.2025.02.025)
Supplement: E-Table 12 [file NIHMS2163178-supplement-E-Table_12.docx]

**E-Table 12.** T2DM Cohort, GLP-1 RA vs. Non-GLP-1 RA Controls at 3 Years Before and After Propensity Score Matching (Non-Arteritic Anterior Ischemic Optic Neuropathy)

|  | **Eligible Cohorts** No. (%) | | | **Cohorts After Matching** No. (%) | | |
| --- | --- | --- | --- | --- | --- | --- |
| **Characteristic Name** | **GLP-1 RA Medications**  **(N = 234941)** | **Non-GLP-1 RA Diabetes Medications ((N = 553423)** | **SMD** | **GLP-1 RA Medications**  **(N= 222787)** | **Non-GLP-1 RA Diabetes Medications (N= 222787)** | **SMD** |
| Current Age, Mean (+/- SD) | 60.9 +/- 13.0 | 67.0 +/- 14.3 | 0.449 | 61.6 +/- 12.6 | 61.1 +/- 14.1 | 0.041 |
| Race |  |  |  |  |  |  |
| *White* | 135713 (57.80%) | 319765 (57.80%) | <0.001 | 129036 (57.90%) | 129522 (58.10%) | 0.004 |
| *Black or African American* | 53532 (22.80%) | 117634 (21.30%) | 0.037 | 50305 (22.60%) | 50639 (22.70%) | 0.004 |
| *Hispanic or Latino* | 27259 (11.60%) | 63318 (11.40%) | 0.005 | 25957 (11.70%) | 25128 (11.30%) | 0.012 |
| Sex |  |  |  |  |  |  |
| *Female* | 130553 (55.60%) | 264061 (47.70%) | 0.158 | 122413 (54.90%) | 122146 (54.80%) | 0.002 |
| BMI |  |  |  |  |  |  |
| *BMI (25-30 kg/m2)* | 63386 (27.00%) | 191507 (34.60%) | 0.166 | 62261 (27.90%) | 63467 (28.50%) | 0.012 |
| *BMI (>30 kg/m2)* | 151186 (64.40%) | 258829 (46.80%) | 0.36 | 139918 (62.80%) | 138976 (62.40%) | 0.009 |
| Essential (primary) hypertension (I10) | 190190 (81.00%) | 414456 (74.90%) | 0.147 | 179350 (80.50%) | 177500 (79.70%) | 0.021 |
| Hyperlipidemia, unspecified (E78.5) | 156040 (66.40%) | 323940 (58.50%) | 0.163 | 146435 (65.70%) | 143012 (64.20%) | 0.032 |
| Sleep apnea (G47.3) | 106521 (45.30%) | 150800 (27.20%) | 0.383 | 95933 (43.10%) | 93643 (42.00%) | 0.021 |
| Other hyperlipidemia (E78.4) | 69589 (29.60%) | 136934 (24.70%) | 0.11 | 64909 (29.10%) | 62531 (28.10%) | 0.024 |
| Atherosclerotic heart disease of native coronary artery (I25.1) | 54789 (23.30%) | 147556 (26.70%) | 0.077 | 53248 (23.90%) | 52248 (23.50%) | 0.011 |
| Chronic kidney disease (CKD) (N18) | 49392 (21.00%) | 136008 (24.60%) | 0.085 | 48035 (21.60%) | 48186 (21.60%) | 0.002 |
| Acute pancreatitis (K85) | 5090 (2.20%) | 16484 (3.00%) | 0.051 | 4998 (2.20%) | 4124 (1.90%) | 0.028 |
| Malignant neoplasm of thyroid gland (C73) | 2073 (0.90%) | 3804 (0.70%) | 0.022 | 1935 (0.90%) | 1684 (0.80%) | 0.013 |
| Other chronic pancreatitis (K86.1) | 1831 (0.80%) | 9018 (1.60%) | 0.078 | 1822 (0.80%) | 1452 (0.70%) | 0.019 |
| Alcohol-induced chronic pancreatitis (K86.0) | 147 (0.10%) | 1487 (0.30%) | 0.051 | 147 (0.10%) | 153 (0.10%) | 0.001 |
| Family history of multiple endocrine neoplasia [MEN] syndrome (Z83.41) | 10 (0.00%) | 28 (0.00%) | 0.001 | 10 (0.00%) | 13 (0.00%) | 0.002 |
| Multiple endocrine neoplasia [MEN] type IIA (E31.22) | 11 (0.00%) | 40 (0.00%) | 0.003 | 10 (0.00%) | 20 (0.00%) | 0.005 |
| Multiple endocrine neoplasia [MEN] type IIB (E31.23) | 10 (0.00%) | 10 (0.00%) | 0.004 | 10 (0.00%) | 10 (0.00%) | <0.001 |
| Sildenafil (136411) | 21136 (9.00%) | 35076 (6.30%) | 0.1 | 19240 (8.60%) | 18810 (8.40%) | 0.007 |
| Tadalafil (358263) | 12637 (5.40%) | 18632 (3.40%) | 0.099 | 11244 (5.00%) | 10506 (4.70%) | 0.015 |
| Amiodarone (703) | 6958 (3.00%) | 24554 (4.40%) | 0.078 | 6882 (3.10%) | 6461 (2.90%) | 0.011 |
| Vardenafil (306674) | 2266 (1.00%) | 4381 (0.80%) | 0.019 | 2105 (0.90%) | 1812 (0.80%) | 0.014 |
| Avanafil (1291301) | 298 (0.10%) | 414 (0.10%) | 0.016 | 269 (0.10%) | 193 (0.10%) | 0.011 |
