# Supplementary material for: The Effect of Semaglutide and GLP-1 RAs on Risk of Nonarteritic Anterior Ischemic Optic Neuropathy
Source: Am J Ophthalmol. Author manuscript; Available in PMC 2026 Apr 25. (PMC13110070; doi:10.1016/j.ajo.2025.02.025)
Supplement: E-Table 16 [file NIHMS2163178-supplement-E-Table_16.docx]

**E-Table 16.** T2DM Cohort, GLP-1 RA vs. Non-GLP-1 RA Controls at 5 Years Before and After Propensity Score Matching (Ischemic Optic Neuropathy)

|  | **Eligible Cohorts** No. (%) | | | **Cohorts After Matching** No. (%) | | |
| --- | --- | --- | --- | --- | --- | --- |
| **Characteristic Name** | **GLP-1 RA Medications**  **(N = 243494)** | **Non-GLP-1 RA Diabetes Medications ((N = 569155)** | **SMD** | **GLP-1 RA Medications**  **(N= 230615)** | **Non-GLP-1 RA Diabetes Medications (N= 230615)** | **SMD** |
| Current Age, Mean (+/- SD) | 60.8 +/- 13.0 | 67.0 +/- 14.3 | 0.448 | 61.6 +/- 12.6 | 61.1 +/- 14.0 | 0.035 |
| Race |  |  |  |  |  |  |
| *White* | 141454 (58.10%) | 330137 (58.00%) | 0.002 | 134270 (58.20%) | 134823 (58.50%) | 0.005 |
| *Black or African American* | 53987 (22.20%) | 118625 (20.80%) | 0.032 | 50759 (22.00%) | 50692 (22.00%) | 0.001 |
| *Hispanic or Latino* | 28679 (11.80%) | 65724 (11.50%) | 0.007 | 27172 (11.80%) | 26103 (11.30%) | 0.015 |
| Sex |  |  |  |  |  |  |
| *Female* | 135378 (55.60%) | 272001 (47.80%) | 0.157 | 126652 (54.90%) | 126497 (54.90%) | 0.001 |
| BMI |  |  |  |  |  |  |
| *BMI (25-30 kg/m2)* | 66592 (27.30%) | 199214 (35.00%) | 0.166 | 65325 (28.30%) | 66828 (29.00%) | 0.014 |
| *BMI (>30 kg/m2)* | 158640 (65.20%) | 268943 (47.30%) | 0.367 | 146635 (63.60%) | 146041 (63.30%) | 0.005 |
| Essential (primary) hypertension (I10) | 197035 (80.90%) | 426474 (74.90%) | 0.145 | 185600 (80.50%) | 183858 (79.70%) | 0.019 |
| Hyperlipidemia, unspecified (E78.5) | 161533 (66.30%) | 333069 (58.50%) | 0.162 | 151396 (65.60%) | 148618 (64.40%) | 0.025 |
| Sleep apnea (G47.3) | 110719 (45.50%) | 155932 (27.40%) | 0.382 | 99473 (43.10%) | 97650 (42.30%) | 0.016 |
| Other hyperlipidemia (E78.4) | 72661 (29.80%) | 142067 (25.00%) | 0.11 | 67643 (29.30%) | 65423 (28.40%) | 0.021 |
| Atherosclerotic heart disease of native coronary artery (I25.1) | 56641 (23.30%) | 151406 (26.60%) | 0.077 | 54946 (23.80%) | 54290 (23.50%) | 0.007 |
| Chronic kidney disease (CKD) (N18) | 51321 (21.10%) | 139841 (24.60%) | 0.083 | 49890 (21.60%) | 50161 (21.80%) | 0.003 |
| Acute pancreatitis (K85) | 5259 (2.20%) | 16874 (3.00%) | 0.051 | 5156 (2.20%) | 4338 (1.90%) | 0.025 |
| Malignant neoplasm of thyroid gland (C73) | 151 (0.10%) | 1509 (0.30%) | 0.05 | 151 (0.10%) | 111 (0.00%) | 0.007 |
| Other chronic pancreatitis (K86.1) | 1926 (0.80%) | 9321 (1.60%) | 0.077 | 1918 (0.80%) | 1494 (0.60%) | 0.021 |
| Alcohol-induced chronic pancreatitis (K86.0) | 2148 (0.90%) | 3928 (0.70%) | 0.022 | 1985 (0.90%) | 1741 (0.80%) | 0.012 |
| Family history of multiple endocrine neoplasia [MEN] syndrome (Z83.41) | 10 (0.00%) | 28 (0.00%) | 0.001 | 10 (0.00%) | 10 (0.00%) | <0.001 |
| Multiple endocrine neoplasia [MEN] type IIA (E31.22) | 14 (0.00%) | 42 (0.00%) | 0.002 | 10 (0.00%) | 21 (0.00%) | 0.006 |
| Multiple endocrine neoplasia [MEN] type IIB (E31.23) | 10 (0.00%) | 10 (0.00%) | 0.004 | 10 (0.00%) | 10 (0.00%) | <0.001 |
| Sildenafil (136411) | 21731 (8.90%) | 35935 (6.30%) | 0.099 | 19820 (8.60%) | 19125 (8.30%) | 0.011 |
| Tadalafil (358263) | 12989 (5.30%) | 19029 (3.30%) | 0.098 | 11553 (5.00%) | 10663 (4.60%) | 0.018 |
| Amiodarone (703) | 7161 (2.90%) | 25118 (4.40%) | 0.078 | 7089 (3.10%) | 6677 (2.90%) | 0.01 |
| Vardenafil (306674) | 2338 (1.00%) | 4475 (0.80%) | 0.019 | 2170 (0.90%) | 1856 (0.80%) | 0.015 |
| Avanafil (1291301) | 305 (0.10%) | 420 (0.10%) | 0.016 | 267 (0.10%) | 213 (0.10%) | 0.007 |
