# Supplementary material for: The Effect of Semaglutide and GLP-1 RAs on Risk of Nonarteritic Anterior Ischemic Optic Neuropathy
Source: Am J Ophthalmol. Author manuscript; Available in PMC 2026 Apr 25. (PMC13110070; doi:10.1016/j.ajo.2025.02.025)
Supplement: E-Table 15 [file NIHMS2163178-supplement-E-Table_15.docx]

**E-Table 15.** T2DM Cohort, GLP-1 RA vs. Non-GLP-1 RA Controls at 3 Years Before and After Propensity Score Matching (Ischemic Optic Neuropathy)

|  | **Eligible Cohorts** No. (%) | | | **Cohorts After Matching** No. (%) | | |
| --- | --- | --- | --- | --- | --- | --- |
| **Characteristic Name** | **GLP-1 RA Medications**  **(N = 235869)** | **Non-GLP-1 RA Diabetes Medications ((N = 556023)** | **SMD** | **GLP-1 RA Medications**  **(N= 223680)** | **Non-GLP-1 RA Diabetes Medications (N= 223680)** | **SMD** |
| Current Age, Mean (+/- SD) | 60.9 +/- 13.0 | 67.1 +/- 14.3 | 0.45 | 61.6 +/- 12.6 | 61.2 +/- 14.1 | 0.037 |
| Race |  |  |  |  |  |  |
| *White* | 136257 (57.80%) | 321403 (57.80%) | 0.001 | 129470 (57.90%) | 130139 (58.20%) | 0.006 |
| *Black or African American* | 53766 (22.80%) | 118169 (21.30%) | 0.037 | 50616 (22.60%) | 50636 (22.60%) | <0.001 |
| *Hispanic or Latino* | 27351 (11.60%) | 63526 (11.40%) | 0.005 | 25938 (11.60%) | 25199 (11.30%) | 0.01 |
| Sex |  |  |  |  |  |  |
| *Female* | 131200 (55.60%) | 265710 (47.80%) | 0.157 | 122952 (55.00%) | 123043 (55.00%) | 0.001 |
| BMI |  |  |  |  |  |  |
| *BMI (25-30 kg/m2)* | 63688 (27.00%) | 192570 (34.60%) | 0.166 | 62527 (28.00%) | 63707 (28.50%) | 0.012 |
| *BMI (>30 kg/m2)* | 151843 (64.40%) | 260204 (46.80%) | 0.359 | 140572 (62.80%) | 139542 (62.40%) | 0.01 |
| Essential (primary) hypertension (I10) | 191050 (81.00%) | 416705 (74.90%) | 0.146 | 180194 (80.60%) | 178321 (79.70%) | 0.021 |
| Hyperlipidemia, unspecified (E78.5) | 156816 (66.50%) | 325834 (58.60%) | 0.163 | 147192 (65.80%) | 144186 (64.50%) | 0.028 |
| Sleep apnea (G47.3) | 107036 (45.40%) | 151700 (27.30%) | 0.383 | 96410 (43.10%) | 94466 (42.20%) | 0.018 |
| Other hyperlipidemia (E78.4) | 69955 (29.70%) | 137815 (24.80%) | 0.11 | 65218 (29.20%) | 63335 (28.30%) | 0.019 |
| Atherosclerotic heart disease of native coronary artery (I25.1) | 55182 (23.40%) | 148672 (26.70%) | 0.077 | 53587 (24.00%) | 52520 (23.50%) | 0.011 |
| Chronic kidney disease (CKD) (N18) | 49734 (21.10%) | 136967 (24.60%) | 0.085 | 48377 (21.60%) | 48454 (21.70%) | 0.001 |
| Acute pancreatitis (K85) | 5117 (2.20%) | 16572 (3.00%) | 0.051 | 5029 (2.20%) | 4323 (1.90%) | 0.022 |
| Malignant neoplasm of thyroid gland (C73) | 2083 (0.90%) | 3829 (0.70%) | 0.022 | 1932 (0.90%) | 1698 (0.80%) | 0.012 |
| Other chronic pancreatitis (K86.1) | 1848 (0.80%) | 9079 (1.60%) | 0.078 | 1840 (0.80%) | 1445 (0.60%) | 0.021 |
| Alcohol-induced chronic pancreatitis (K86.0) | 147 (0.10%) | 1490 (0.30%) | 0.051 | 147 (0.10%) | 184 (0.10%) | 0.006 |
| Family history of multiple endocrine neoplasia [MEN] syndrome (Z83.41) | 10 (0.00%) | 28 (0.00%) | 0.001 | 10 (0.00%) | 10 (0.00%) | <0.001 |
| Multiple endocrine neoplasia [MEN] type IIA (E31.22) | 11 (0.00%) | 40 (0.00%) | 0.003 | 10 (0.00%) | 15 (0.00%) | 0.003 |
| Multiple endocrine neoplasia [MEN] type IIB (E31.23) | 10 (0.00%) | 10 (0.00%) | 0.004 | 10 (0.00%) | 10 (0.00%) | <0.001 |
| Sildenafil (136411) | 21198 (9.00%) | 35189 (6.30%) | 0.1 | 19343 (8.60%) | 18691 (8.40%) | 0.01 |
| Tadalafil (358263) | 12667 (5.40%) | 18679 (3.40%) | 0.099 | 11302 (5.10%) | 10565 (4.70%) | 0.015 |
| Amiodarone (703) | 6998 (3.00%) | 24716 (4.40%) | 0.078 | 6933 (3.10%) | 6636 (3.00%) | 0.008 |
| Vardenafil (306674) | 2275 (1.00%) | 4401 (0.80%) | 0.019 | 2120 (0.90%) | 1816 (0.80%) | 0.015 |
| Avanafil (1291301) | 298 (0.10%) | 416 (0.10%) | 0.016 | 274 (0.10%) | 184 (0.10%) | 0.013 |
