# Supplementary material for: The Effect of Semaglutide and GLP-1 RAs on Risk of Nonarteritic Anterior Ischemic Optic Neuropathy
Source: Am J Ophthalmol. Author manuscript; Available in PMC 2026 Apr 25. (PMC13110070; doi:10.1016/j.ajo.2025.02.025)
Supplement: E-Table 7 [file NIHMS2163178-supplement-E-Table_7.docx]

**E-Table 7.** High BMI Cohort, Semaglutide vs. Non-GLP-1 RA Controls at 1 Year Before and After Propensity Score Matching (Non-Arteritic Anterior Ischemic Optic Neuropathy)

|  | **Eligible Cohorts** No. (%) | | | **Cohorts After Matching** No. (%) | | |
| --- | --- | --- | --- | --- | --- | --- |
| **Characteristic Name** | **semaglutide**  **(N = 95,706)** | **Non-GLP-1 RA Diabetes Medications (N =126,435)** | **SMD** | **semaglutide (N= 57,913)** | **Non-GLP-1 RA Diabetes Medications (N=57,913)** | **SMD** |
| Current Age, Mean (+/- SD) | 95706 (100.00%) | 126435 (100.00%) | 0.291 | 57913 (100.00%) | 57913 (100.00%) | 0.018 |
| Race |  |  |  |  |  |  |
| *White* | 58405 (61.00%) | 90126 (71.30%) | 0.218 | 37742 (65.20%) | 38114 (65.80%) | 0.014 |
| *Black or African American* | 18702 (19.50%) | 16469 (13.00%) | 0.177 | 9648 (16.70%) | 9565 (16.50%) | 0.004 |
| *Hispanic or Latino* | 10344 (10.80%) | 11034 (8.70%) | 0.07 | 5775 (10.00%) | 5531 (9.60%) | 0.014 |
| Sex |  |  |  |  |  |  |
| *Female* | 59784 (62.50%) | 83716 (66.20%) | 0.078 | 37834 (65.30%) | 39173 (67.60%) | 0.049 |
| BMI |  |  |  |  |  |  |
| *BMI (25-30 kg/m2)* | 35961 (37.60%) | 73944 (58.50%) | 0.012 | 25696 (44.40%) | 24930 (43.00%) | 0.027 |
| *BMI (>30 kg/m2)* | 87995 (91.90%) | 91473 (72.30%) | 0.001 | 50974 (88.00%) | 52170 (90.10%) | 0.066 |
| Essential (primary) hypertension (I10) | 68888 (72.00%) | 61631 (48.70%) | 0.489 | 36470 (63.00%) | 36517 (63.10%) | 0.002 |
| Hyperlipidemia, unspecified (E78.5) | 56269 (58.80%) | 45787 (36.20%) | 0.464 | 28515 (49.20%) | 28281 (48.80%) | 0.008 |
| Sleep apnea (G47.3) | 49592 (51.80%) | 47481 (37.60%) | 0.29 | 27924 (48.20%) | 29383 (50.70%) | 0.05 |
| Other hyperlipidemia (E78.4) | 27159 (28.40%) | 19803 (15.70%) | 0.311 | 13016 (22.50%) | 12684 (21.90%) | 0.014 |
| Atherosclerotic heart disease of native coronary artery (I25.1) | 17705 (18.50%) | 14640 (11.60%) | 0.195 | 9192 (15.90%) | 8748 (15.10%) | 0.021 |
| Chronic kidney disease (CKD) (N18) | 15810 (16.50%) | 12720 (10.10%) | 0.191 | 8068 (13.90%) | 7489 (12.90%) | 0.029 |
| Acute pancreatitis (K85) | 1739 (1.80%) | 2887 (2.30%) | 0.033 | 1182 (2.00%) | 1183 (2.00%) | <0.001 |
| Malignant neoplasm of thyroid gland (C73) | 943 (1.00%) | 1035 (0.80%) | 0.018 | 562 (1.00%) | 518 (0.90%) | 0.008 |
| Other chronic pancreatitis (K86.1) | 662 (0.70%) | 1367 (1.10%) | 0.042 | 528 (0.90%) | 525 (0.90%) | 0.001 |
| Alcohol-induced chronic pancreatitis (K86.0) | 34 (0.00%) | 293 (0.20%) | 0.054 | 34 (0.10%) | 28 (0.00%) | 0.004 |
| Family history of multiple endocrine neoplasia [MEN] syndrome (Z83.41) | 10 (0.00%) | 14 (0.00%) | 0.001 | 10 (0.00%) | 10 (0.00%) | <0.001 |
| Multiple endocrine neoplasia [MEN] type IIA (E31.22) | 10 (0.00%) | 18 (0.00%) | 0.003 | 10 (0.00%) | 10 (0.00%) | <0.001 |
| Multiple endocrine neoplasia [MEN] type IIB (E31.23) | 0 (0.00%) | 10 (0.00%) | 0.013 | 0 (0.00%) | 10 (0.00%) | 0.019 |
| Type 2 diabetes mellitus [T2DM] (E11) | 61655 (64.40%) | 23682 (18.70%) | 1.046 | 24103 (41.60%) | 23027 (39.80%) | 0.038 |
| Sildenafil (136411) | 7750 (8.10%) | 6908 (5.50%) | 0.105 | 3909 (6.70%) | 3518 (6.10%) | 0.028 |
| Tadalafil (358263) | 5024 (5.20%) | 4317 (3.40%) | 0.09 | 2501 (4.30%) | 2300 (4.00%) | 0.017 |
| Amiodarone (703) | 2260 (2.40%) | 2263 (1.80%) | 0.04 | 1303 (2.20%) | 1154 (2.00%) | 0.018 |
| Vardenafil (306674) | 765 (0.80%) | 577 (0.50%) | 0.043 | 332 (0.60%) | 323 (0.60%) | 0.002 |
| Avanafil (1291301) | 115 (0.10%) | 81 (0.10%) | 0.018 | 51 (0.10%) | 43 (0.10%) | 0.005 |
