# Supplementary material for: The Effect of Semaglutide and GLP-1 RAs on Risk of Nonarteritic Anterior Ischemic Optic Neuropathy
Source: Am J Ophthalmol. Author manuscript; Available in PMC 2026 Apr 25. (PMC13110070; doi:10.1016/j.ajo.2025.02.025)
Supplement: E-Table 10 [file NIHMS2163178-supplement-E-Table_10.docx]

**E-Table 10.** High BMI Cohort, Semaglutide vs. Non-GLP-1 RA Controls at 2 Years Before and After Propensity Score Matching (Ischemic Optic Neuropathy)

|  | **Eligible Cohorts** No. (%) | | | **Cohorts After Matching** No. (%) | | |
| --- | --- | --- | --- | --- | --- | --- |
| **Characteristic Name** | **semaglutide**  **(N = 95,953)** | **Non-GLP-1 RA Diabetes Medications (N = 126,782)** | **SMD** | **semaglutide (N= 58,088)** | **Non-GLP-1 RA Diabetes Medications (N= 58,088)** | **SMD** |
| Current Age, Mean (+/- SD) | 95953 (100.00%) | 126782 (100.00%) | 0.29 | 58088 (100.00%) | 58088 (100.00%) | 0.017 |
| Race |  |  |  |  |  |  |
| *White* | 58560 (61.00%) | 90371 (71.30%) | 0.218 | 37745 (65.00%) | 38277 (65.90%) | 0.019 |
| *Black or African American* | 18756 (19.50%) | 16524 (13.00%) | 0.177 | 9749 (16.80%) | 9583 (16.50%) | 0.008 |
| *Hispanic or Latino* | 10360 (10.80%) | 11065 (8.70%) | 0.07 | 5796 (10.00%) | 5604 (9.60%) | 0.011 |
| Sex |  |  |  |  |  |  |
| *Female* | 59960 (62.50%) | 83980 (66.20%) | 0.078 | 37862 (65.20%) | 39337 (67.70%) | 0.054 |
| BMI |  |  |  |  |  |  |
| *BMI (25-30 kg/m2)* | 36068 (37.60%) | 74161 (58.50%) | 0.428 | 25809 (44.40%) | 24993 (43.00%) | 0.028 |
| *BMI (>30 kg/m2)* | 88220 (91.90%) | 91738 (72.40%) | 0.529 | 51105 (88.00%) | 52318 (90.10%) | 0.067 |
| Essential (primary) hypertension (I10) | 69109 (72.00%) | 61875 (48.80%) | 0.489 | 36578 (63.00%) | 36648 (63.10%) | 0.002 |
| Hyperlipidemia, unspecified (E78.5) | 56463 (58.80%) | 45983 (36.30%) | 0.464 | 28031 (48.30%) | 29674 (51.10%) | 0.057 |
| Sleep apnea (G47.3) | 49742 (51.80%) | 47650 (37.60%) | 0.29 | 10 (0.00%) | 10 (0.00%) | <0.001 |
| Other hyperlipidemia (E78.4) | 27260 (28.40%) | 19898 (15.70%) | 0.31 | 10 (0.00%) | 10 (0.00%) | <0.001 |
| Atherosclerotic heart disease of native coronary artery (I25.1) | 17802 (18.60%) | 14753 (11.60%) | 0.194 | 0 (0.00%) | 10 (0.00%) | 0.019 |
| Chronic kidney disease (CKD) (N18) | 15885 (16.60%) | 12807 (10.10%) | 0.191 | 572 (1.00%) | 528 (0.90%) | 0.008 |
| Acute pancreatitis (K85) | 1743 (1.80%) | 2896 (2.30%) | 0.033 | 8098 (13.90%) | 7583 (13.10%) | 0.026 |
| Malignant neoplasm of thyroid gland (C73) | 945 (1.00%) | 1043 (0.80%) | 0.017 | 1217 (2.10%) | 1178 (2.00%) | 0.005 |
| Other chronic pancreatitis (K86.1) | 665 (0.70%) | 1372 (1.10%) | 0.041 | 34 (0.10%) | 30 (0.10%) | 0.003 |
| Alcohol-induced chronic pancreatitis (K86.0) | 34 (0.00%) | 293 (0.20%) | 0.054 | 517 (0.90%) | 533 (0.90%) | 0.003 |
| Family history of multiple endocrine neoplasia [MEN] syndrome (Z83.41) | 10 (0.00%) | 14 (0.00%) | 0.001 | 13089 (22.50%) | 12736 (21.90%) | 0.015 |
| Multiple endocrine neoplasia [MEN] type IIA (E31.22) | 10 (0.00%) | 18 (0.00%) | 0.003 | 28577 (49.20%) | 28355 (48.80%) | 0.008 |
| Multiple endocrine neoplasia [MEN] type IIB (E31.23) | 0 (0.00%) | 10 (0.00%) | 0.013 | 9280 (16.00%) | 8858 (15.20%) | 0.02 |
| Type 2 Diabetes Mellitus [T2DM] (E11) | 61862 (64.50%) | 23808 (18.80%) | 1.046 | 24255 (41.80%) | 23147 (39.80%) | 0.039 |
| Sildenafil (136411) | 7763 (8.10%) | 6925 (5.50%) | 0.105 | 3796 (6.50%) | 3517 (6.10%) | 0.02 |
| Tadalafil (358263) | 5032 (5.20%) | 4327 (3.40%) | 0.09 | 2460 (4.20%) | 2334 (4.00%) | 0.011 |
| Amiodarone (703) | 2272 (2.40%) | 2282 (1.80%) | 0.04 | 1275 (2.20%) | 1169 (2.00%) | 0.013 |
| Vardenafil (306674) | 767 (0.80%) | 578 (0.50%) | 0.043 | 358 (0.60%) | 331 (0.60%) | 0.006 |
| Avanafil (1291301) | 115 (0.10%) | 82 (0.10%) | 0.018 | 45 (0.10%) | 41 (0.10%) | 0.003 |
