# Supplementary material for: The Effect of Semaglutide and GLP-1 RAs on Risk of Nonarteritic Anterior Ischemic Optic Neuropathy
Source: Am J Ophthalmol. Author manuscript; Available in PMC 2026 Apr 25. (PMC13110070; doi:10.1016/j.ajo.2025.02.025)
Supplement: E-Table 11 [file NIHMS2163178-supplement-E-Table_11.docx]

**E-Table 11.** T2DM Cohort, GLP-1 RA vs. Non-GLP-1 RA Controls at 1 Year Before and After Propensity Score Matching (Non-Arteritic Anterior Ischemic Optic Neuropathy)

|  | **Eligible Cohorts** No. (%) | | | **Cohorts After Matching** No. (%) | | |
| --- | --- | --- | --- | --- | --- | --- |
| **Characteristic Name** | **GLP-1 RA Medications**  **(N = 234879)** | **Non-GLP-1 RA Diabetes Medications ((N = 554024)** | **SMD** | **GLP-1 RA Medications**  **(N= 222580)** | **Non-GLP-1 RA Diabetes Medications (N= 222580)** | **SMD** |
| Current Age, Mean (+/- SD) | 60.8 +/- 13.0 | 66.9 +/- 14.4 | 0.448 | 61.5 +/- 12.6 | 61.1 +/- 14.1 | 0.033 |
| Race |  |  |  |  |  |  |
| *White* | 139291 (59.30%) | 325325 (58.70%) | 0.012 | 132072 (59.30%) | 132606 (59.60%) | 0.005 |
| *Black or African American* | 49807 (21.20%) | 112301 (20.30%) | 0.023 | 46993 (21.10%) | 47216 (21.20%) | 0.002 |
| *Hispanic or Latino* | 26700 (11.40%) | 63153 (11.40%) | 0.001 | 25379 (11.40%) | 24730 (11.10%) | 0.009 |
| Sex |  |  |  |  |  |  |
| *Female* | 130422 (55.50%) | 264005 (47.70%) | 0.158 | 122163 (54.90%) | 122449 (55.00%) | 0.003 |
| BMI |  |  |  |  |  |  |
| *BMI (25-30 kg/m2)* | 62799 (26.70%) | 192223 (34.70%) | 0.173 | 61691 (27.70%) | 62941 (28.30%) | 0.013 |
| *BMI (>30 kg/m2)* | 152525 (64.90%) | 261541 (47.20%) | 0.363 | 141158 (63.40%) | 140405 (63.10%) | 0.007 |
| Essential (primary) hypertension (I10) | 190512 (81.10%) | 416329 (75.10%) | 0.145 | 179535 (80.70%) | 177891 (79.90%) | 0.019 |
| Hyperlipidemia, unspecified (E78.5) | 156875 (66.80%) | 326144 (58.90%) | 0.164 | 147114 (66.10%) | 144142 (64.80%) | 0.028 |
| Sleep apnea (G47.3) | 108209 (46.10%) | 153328 (27.70%) | 0.388 | 97455 (43.80%) | 95668 (43.00%) | 0.016 |
| Other hyperlipidemia (E78.4) | 68697 (29.20%) | 136629 (24.70%) | 0.104 | 64133 (28.80%) | 61989 (27.90%) | 0.021 |
| Atherosclerotic heart disease of native coronary artery (I25.1) | 55092 (23.50%) | 148507 (26.80%) | 0.077 | 53512 (24.00%) | 52249 (23.50%) | 0.013 |
| Chronic kidney disease (CKD) (N18) | 49221 (21.00%) | 136386 (24.60%) | 0.087 | 47897 (21.50%) | 47878 (21.50%) | <0.001 |
| Acute pancreatitis (K85) | 5052 (2.20%) | 16407 (3.00%) | 0.051 | 4940 (2.20%) | 4132 (1.90%) | 0.026 |
| Malignant neoplasm of thyroid gland (C73) | 2098 (0.90%) | 3815 (0.70%) | 0.023 | 1939 (0.90%) | 1736 (0.80%) | 0.01 |
| Other chronic pancreatitis (K86.1) | 1837 (0.80%) | 9012 (1.60%) | 0.077 | 1830 (0.80%) | 1407 (0.60%) | 0.022 |
| Alcohol-induced chronic pancreatitis (K86.0) | 145 (0.10%) | 1465 (0.30%) | 0.05 | 144 (0.10%) | 169 (0.10%) | 0.004 |
| Family history of multiple endocrine neoplasia [MEN] syndrome (Z83.41) | 10 (0.00%) | 28 (0.00%) | 0.001 | 10 (0.00%) | 15 (0.00%) | 0.003 |
| Multiple endocrine neoplasia [MEN] type IIA (E31.22) | 14 (0.00%) | 40 (0.00%) | 0.002 | 11 (0.00%) | 26 (0.00%) | 0.007 |
| Multiple endocrine neoplasia [MEN] type IIB (E31.23) | 10 (0.00%) | 10 (0.00%) | 0.004 | 10 (0.00%) | 10 (0.00%) | <0.001 |
| Sildenafil (136411) | 20784 (8.80%) | 34765 (6.30%) | 0.097 | 18885 (8.50%) | 18353 (8.20%) | 0.009 |
| Tadalafil (358263) | 12528 (5.30%) | 18449 (3.30%) | 0.099 | 11061 (5.00%) | 10479 (4.70%) | 0.012 |
| Amiodarone (703) | 6997 (3.00%) | 24776 (4.50%) | 0.079 | 6916 (3.10%) | 6588 (3.00%) | 0.009 |
| Vardenafil (306674) | 2222 (0.90%) | 4315 (0.80%) | 0.018 | 2039 (0.90%) | 1793 (0.80%) | 0.012 |
| Avanafil (1291301) | 295 (0.10%) | 411 (0.10%) | 0.016 | 266 (0.10%) | 183 (0.10%) | 0.012 |
