# Supplementary material for: The Effect of Semaglutide and GLP-1 RAs on Risk of Nonarteritic Anterior Ischemic Optic Neuropathy
Source: Am J Ophthalmol. Author manuscript; Available in PMC 2026 Apr 25. (PMC13110070; doi:10.1016/j.ajo.2025.02.025)
Supplement: E-Table 6 [file NIHMS2163178-supplement-E-Table_6.docx]

**E-Table 6.** T2DM Cohort, Semaglutide vs. Non-GLP-1 RA Controls at 5 Years Before and After Propensity Score Matching (Ischemic Optic Neuropathy)

|  | **Eligible Cohorts** No. (%) | | | **Cohorts After Matching** No. (%) | | |
| --- | --- | --- | --- | --- | --- | --- |
| **Characteristic Name** | **semaglutide**  **(N = 131447)** | **Non-GLP-1 RA Diabetes Medications ((N = 578052)** | **SMD** | **semaglutide (N= 130446)** | **Non-GLP-1 RA Diabetes Medications (N= 130446)** | **SMD** |
| Current Age, Mean (+/- SD) | 60.1 (+/- 12.7) | 67.0 (+/- 14.3) | 0.513 | 60.2 +/- 12.6 | 59.9 +/- 13.7 | 0.027 |
| Race |  |  |  |  |  |  |
| *White* | 79368 (60.38%) | 337967 (58.47%) | 0.039 | 78701 (60.33%) | 79098 (60.64%) | 0.006 |
| *Black or African American* | 26707 (20.32%) | 117384 (20.31%) | 0.000 | 26537 (20.34%) | 26651 (20.43%) | 0.002 |
| *Hispanic or Latino* | 13344 (10.15%) | 70307 (12.16%) | 0.064 | 13285 (10.18%) | 12460 (9.55%) | 0.021 |
| Sex |  |  |  |  |  |  |
| *Female* | 74359 (56.57%) | 275830 (47.72%) | 0.178 | 73687 (56.49%) | 74313 (56.97%) | 0.01 |
| BMI |  |  |  |  |  |  |
| *BMI (25-30 kg/m2)* | 36116 (27.48%) | 210286 (36.38%) | 0.192 | 36047 (27.63%) | 36583 (28.05%) | 0.009 |
| *BMI (>30 kg/m2)* | 90150 (68.58%) | 280319 (48.49%) | 0.417 | 89181 (68.37%) | 88923 (68.17%) | 0.004 |
| Essential (primary) hypertension (I10) | 108299 (82.39%) | 433937 (75.07%) | 0.180 | 107385 (82.32%) | 106982 (82.01%) | 0.008 |
| Hyperlipidemia, unspecified (E78.5) | 90204 (68.62%) | 341430 (59.07%) | 0.200 | 89335 (68.48%) | 87988 (67.45%) | 0.022 |
| Sleep apnea (G47.3) | 66814 (50.83%) | 160075 (27.69%) | 0.488 | 65850 (50.48%) | 64750 (49.64%) | 0.017 |
| Other hyperlipidemia (E78.4) | 39038 (29.70%) | 138065 (23.89%) | 0.132 | 38646 (29.63%) | 37233 (28.54%) | 0.024 |
| Atherosclerotic heart disease of native coronary artery (I25.1) | 31909 (24.28%) | 157892 (27.32%) | 0.070 | 31825 (24.40%) | 31076 (23.82%) | 0.013 |
| Chronic kidney disease (CKD) (N18) | 27425 (20.86%) | 145364 (25.15%) | 0.102 | 27345 (20.96%) | 27342 (20.96%) | 0.000 |
| Acute pancreatitis (K85) | 2780 (2.12%) | 17300 (2.99%) | 0.056 | 2774 (2.13%) | 2323 (1.78%) | 0.025 |
| Malignant neoplasm of thyroid gland (C73) | 1338 (1.02%) | 3972 (0.69%) | 0.036 | 1320 (1.01%) | 1116 (0.86%) | 0.016 |
| Other chronic pancreatitis (K86.1) | 1013 (0.77%) | 9474 (1.64%) | 0.080 | 1013 (0.78%) | 784 (0.60%) | 0.021 |
| Alcohol-induced chronic pancreatitis (K86.0) | 61 (0.05%) | 1528 (0.26%) | 0.055 | 61 (0.05%) | 50 (0.04%) | 0.004 |
| Family history of multiple endocrine neoplasia [MEN] syndrome (Z83.41) | 10 (0.01%) | 28 (0.01%) | 0.004 | 10 (0.01%) | 10 (0.01%) | 0.000 |
| Multiple endocrine neoplasia [MEN] type IIA (E31.22) | 10 (0.01%) | 42 (0.01%) | 0.000 | 10 (0.01%) | 10 (0.01%) | 0.000 |
| Multiple endocrine neoplasia [MEN] type IIB (E31.23) | 0 (0.00%) | 10 (0.00%) | 0.006 | 0 (0.00%) | 0 (0.00%) | 0.008 |
| Sildenafil (136411) | 12103 (9.21%) | 35599 (6.16%) | 0.115 | 11885 (9.11%) | 11295 (8.66%) | 0.016 |
| Tadalafil (358263) | 7587 (5.77%) | 18807 (3.25%) | 0.122 | 7395 (5.67%) | 6659 (5.11%) | 0.025 |
| Amiodarone (703) | 4150 (3.16%) | 27304 (4.72%) | 0.081 | 4143 (3.18%) | 3967 (3.04%) | 0.008 |
| Vardenafil (306674) | 1203 (0.92%) | 4367 (0.76%) | 0.018 | 1191 (0.91%) | 943 (0.72%) | 0.021 |
| Avanafil (1291301) | 185 (0.14%) | 417 (0.07%) | 0.021 | 183 (0.14%) | 127 (0.10%) | 0.012 |
