# Supplementary material for: The Effect of Semaglutide and GLP-1 RAs on Risk of Nonarteritic Anterior Ischemic Optic Neuropathy
Source: Am J Ophthalmol. Author manuscript; Available in PMC 2026 Apr 25. (PMC13110070; doi:10.1016/j.ajo.2025.02.025)
Supplement: E-Table 5 [file NIHMS2163178-supplement-E-Table_5.docx]

**E-Table 5.** T2DM Cohort, Semaglutide vs. Non-GLP-1 RA Controls at 3 Years Before and After Propensity Score Matching (Ischemic Optic Neuropathy)

|  | **Eligible Cohorts** No. (%) | | | **Cohorts After Matching** No. (%) | | |
| --- | --- | --- | --- | --- | --- | --- |
| **Characteristic Name** | **semaglutide**  **(N = 131447)** | **Non-GLP-1 RA Diabetes Medications ((N = 578052)** | **SMD** | **semaglutide (N= 130446)** | **Non-GLP-1 RA Diabetes Medications (N= 130446)** | **SMD** |
| Current Age, Mean (+/- SD) |  | 67.0 (+/- 14.3) | 0.513 | 60.2 +/- 12.6 | 59.9 +/- 13.7 | 0.027 |
| Race |  |  |  |  |  |  |
| *White* | 79368 (60.40%) | 337967 (58.50%) | 0.039 | 78701 (60.30%) | 79098 (60.60%) | 0.006 |
| *Black or African American* | 26707 (20.30%) | 117384 (20.30%) | <0.001 | 26537 (20.30%) | 26651 (20.40%) | 0.002 |
| *Hispanic or Latino* | 13344 (10.20%) | 70307 (12.20%) | 0.064 | 13285 (10.20%) | 12460 (9.60%) | 0.021 |
| Sex |  |  |  |  |  |  |
| *Female* | 74359 (56.60%) | 275830 (47.70%) | 0.178 | 73687 (56.50%) | 74313 (57.00%) | 0.01 |
| BMI |  |  |  |  |  |  |
| *BMI (25-30 kg/m2)* | 36116 (27.5%) | 210286 (36.4%) | 0.193 | 36047 (27.60%) | 36583 (28.00%) | 0.009 |
| *BMI (>30 kg/m2)* | 90150 (68.6%) | 280319 (48.5%%) | 0.417 | 89181 (68.40%) | 88923 (68.20%) | 0.004 |
| Essential (primary) hypertension (I10) | 108299 (82.4%) | 433937 (75.1%) | 0.18 | 107385 (82.30%) | 106982 (82.00%) | 0.008 |
| Hyperlipidemia, unspecified (E78.5) | 86887 (68.43%) | 332810 (59.04%) | 0.196 | 89335 (68.50%) | 87988 (67.50%) | 0.022 |
| Sleep apnea (G47.3) | 66814 (50.8%) | 160075 (27.7%) | 0.488 | 65850 (50.50%) | 64750 (49.60%) | 0.017 |
| Other hyperlipidemia (E78.4) | 39038 (29.7%) | 138065 (23.9%) | 0.132 | 38646 (29.60%) | 37233 (28.50%) | 0.024 |
| Atherosclerotic heart disease of native coronary artery (I25.1) | 31909 (24.3%) | 157892 (27.3%) | 0.07 | 31825 (24.40%) | 31076 (23.80%) | 0.013 |
| Chronic kidney disease (CKD) (N18) | 27425 (20.9%) | 145364 (25.1%) | 0.102 | 27345 (21.00%) | 27342 (21.00%) | <0.001 |
| Acute pancreatitis (K85) | 2780 (2.1%) | 17300 (3.0%) | 0.056 | 2774 (2.10%) | 2323 (1.80%) | 0.025 |
| Malignant neoplasm of thyroid gland (C73) | 1338 (1.0%) | 3972 (0.7%) | 0.036 | 1320 (1.00%) | 1116 (0.90%) | 0.016 |
| Other chronic pancreatitis (K86.1) | 1013 (0.8%) | 9474 (1.6%) | 0.08 | 1013 (0.80%) | 784 (0.60%) | 0.021 |
| Alcohol-induced chronic pancreatitis (K86.0) | 61 (0.0%) | 1528 (0.3%) | 0.055 | 61 (0.00%) | 50 (0.00%) | 0.004 |
| Family history of multiple endocrine neoplasia [MEN] syndrome (Z83.41) | 10 (0.0%) | 28 (0.0%) | 0.004 | 10 (0.00%) | 10 (0.00%) | <0.001 |
| Multiple endocrine neoplasia [MEN] type IIA (E31.22) | 10 (0.0%) | 42 (0.0%) | <0.001 | 10 (0.00%) | 10 (0.00%) | <0.001 |
| Multiple endocrine neoplasia [MEN] type IIB (E31.23) | 0 (0.00%) | 10 (0.00%) | 0.006 | 0 (0.00%) | 0 (0.00%) | 0.008 |
| Sildenafil (136411) | 12103 (9.20%) | 35599 (6.20%) | 0.115 | 11885 (9.10%) | 11295 (8.70%) | 0.016 |
| Tadalafil (358263) | 7587 (5.80%) | 18807 (3.30%) | 0.122 | 7395 (5.70%) | 6659 (5.10%) | 0.025 |
| Amiodarone (703) | 4150 (3.20%) | 27304 (4.70%) | 0.081 | 4143 (3.20%) | 3967 (3.00%) | 0.008 |
| Vardenafil (306674) | 1203 (0.90%) | 4367 (0.80%) | 0.018 | 1191 (0.90%) | 943 (0.70%) | 0.021 |
| Avanafil (1291301) | 185 (0.10%) | 417 (0.10%) | 0.021 | 183 (0.10%) | 127 (0.10%) | 0.012 |
