# Supplementary material for: The Effect of Semaglutide and GLP-1 RAs on Risk of Nonarteritic Anterior Ischemic Optic Neuropathy
Source: Am J Ophthalmol. Author manuscript; Available in PMC 2026 Apr 25. (PMC13110070; doi:10.1016/j.ajo.2025.02.025)
Supplement: E-Table 3 [file NIHMS2163178-supplement-E-Table_3.docx]

**E-Table 3.** T2DM Cohort, Semaglutide vs. Non-GLP-1 RA Controls at 5 Years Before and After Propensity Score Matching (Non-Arteritic Anterior Ischemic Optic Neuropathy)

|  | **Eligible Cohorts** No. (%) | | | **Cohorts After Matching** No. (%) | | |
| --- | --- | --- | --- | --- | --- | --- |
| **Characteristic Name** | **semaglutide**  **(N = 119143)** | **Non-GLP-1 RA Diabetes Medications ((N = 535098)** | **SMD** | **semaglutide (N= 118277)** | **Non-GLP-1 RA Diabetes Medications (N= 118277)** | **SMD** |
| Current Age, Mean (+/- SD) | 59.9 +/- 12.7 | 67.0 +/- 14.3 | 0.524 | 60.2 +/- 12.6 | 59.8 +/- 13.7 | 0.030 |
| Race |  |  |  |  |  |  |
| *White* | 68299 (57.30%) | 301342 (56.30%) | 0.02 | 67780 (57.30%) | 67837 (57.40%) | 0.001 |
| *Black or African American* | 26241 (22.00%) | 114679 (21.40%) | 0.014 | 26043 (22.00%) | 26308 (22.20%) | 0.005 |
| *Hispanic or Latino* | 12075 (10.10%) | 65158 (12.20%) | 0.065 | 12030 (10.20%) | 11294 (9.50%) | 0.021 |
| Sex |  |  |  |  |  |  |
| *Female* | 67297 (56.50%) | 255083 (47.70%) | 0.177 | 66682 (56.40%) | 67199 (56.80%) | 0.009 |
| BMI |  |  |  |  |  |  |
| *BMI (25-30 kg/m2)* | 34305 (28.80%) | 194139 (36.30%) | 0.184 | 34245 (29.00%) | 34948 (29.50%) | 0.012 |
| *BMI (>30 kg/m2)* | 86015 (72.20%) | 260680 (48.70%) | 0.466 | 85159 (72.00%) | 84942 (71.80%) | 0.001 |
| Essential (primary) hypertension (I10) | 97170 (81.60%) | 397863 (74.40%) | 0.174 | 96376 (81.50%) | 95982 (81.20%) | 0.009 |
| Hyperlipidemia, unspecified (E78.5) | 79865 (67.00%) | 309665 (57.90%) | 0.19 | 79144 (66.90%) | 77621 (65.60%) | 0.027 |
| Sleep apnea (G47.3) | 59492 (49.90%) | 143931 (26.90%) | 0.487 | 58646 (49.60%) | 57666 (48.80%) | 0.017 |
| Other hyperlipidemia (E78.4) | 37720 (31.70%) | 137690 (25.70%) | 0.131 | 37370 (31.60%) | 36049 (30.50%) | 0.024 |
| Atherosclerotic heart disease of native coronary artery (I25.1) | 27199 (22.80%) | 137981 (25.80%) | 0.069 | 27142 (22.90%) | 26377 (22.30%) | 0.015 |
| Chronic kidney disease (CKD) (N18) | 24522 (20.60%) | 130007 (24.30%) | 0.089 | 24459 (20.70%) | 24498 (20.70%) | 0.001 |
| Acute pancreatitis (K85) | 2449 (2.10%) | 15624 (2.90%) | 0.056 | 2445 (2.10%) | 1965 (1.70%) | 0.03 |
| Malignant neoplasm of thyroid gland (C73) | 1206 (1.00%) | 3684 (0.70%) | 0.035 | 1194 (1.00%) | 960 (0.80%) | 0.021 |
| Other chronic pancreatitis (K86.1) | 918 (0.80%) | 8779 (1.60%) | 0.08 | 917 (0.80%) | 679 (0.60%) | 0.025 |
| Alcohol-induced chronic pancreatitis (K86.0) | 55 (0.00%) | 1445 (0.30%) | 0.056 | 55 (0.00%) | 67 (0.10%) | 0.004 |
| Family history of multiple endocrine neoplasia [MEN] syndrome (Z83.41) | 10 (0.00%) | 26 (0.00%) | 0.004 | 10 (0.00%) | 10 (0.00%) | <0.001 |
| Multiple endocrine neoplasia [MEN] type IIA (E31.22) | 10 (0.00%) | 40 (0.00%) | 0.001 | 10 (0.00%) | 10 (0.00%) | <0.001 |
| Multiple endocrine neoplasia [MEN] type IIB (E31.23) | 0 (0.00%) | 10 (0.00%) | 0.006 | 0 (0.00%) | 0 (0.00%) | -- |
| Sildenafil (136411) | 11268 (9.50%) | 34330 (6.40%) | 0.03 | 11107 (9.40%) | 10455 (8.80%) | 0.028 |
| Tadalafil (358263) | 7104 (6.00%) | 18299 (3.40%) | 0.028 | 6957 (5.90%) | 6185 (5.20%) | 0.016 |
| Amiodarone (703) | 3599 (3.00%) | 22995 (4.30%) | 0.021 | 3591 (3.00%) | 3356 (2.80%) | 0.03 |
| Vardenafil (306674) | 1137 (1.00%) | 4305 (0.80%) | 0.016 | 1131 (1.00%) | 928 (0.80%) | 0.021 |
| Avanafil (1291301) | 179 (0.20%) | 406 (0.10%) | 0.011 | 177 (0.10%) | 90 (0.10%) | 0.011 |
