# Supplementary material for: The Effect of Semaglutide and GLP-1 RAs on Risk of Nonarteritic Anterior Ischemic Optic Neuropathy
Source: Am J Ophthalmol. Author manuscript; Available in PMC 2026 Apr 25. (PMC13110070; doi:10.1016/j.ajo.2025.02.025)
Supplement: E-Table 2 [file NIHMS2163178-supplement-E-Table_2.docx]

**E-Table 2.** T2DM Cohort, Semaglutide vs. Non-GLP-1 RA Controls at 3 Years Before and After Propensity Score Matching (Non-Arteritic Anterior Ischemic Optic Neuropathy)

|  | **Eligible Cohorts** No. (%) | | | **Cohorts After Matching** No. (%) | | |
| --- | --- | --- | --- | --- | --- | --- |
| **Characteristic Name** | **semaglutide**  **(N = 112451)** | **Non-GLP-1 RA Diabetes Medications ((N = 504490)** | **SMD** | **semaglutide (N= 111656)** | **Non-GLP-1 RA Diabetes Medications (N= 111656)** | **SMD** |
| Current Age, Mean (+/- SD) | 59.9 +/- 12.7 | 67.0 +/- 14.3 | 0.524 | 60.1 +/- 12.6 | 59.7 +/- 13.8 | 0.025 |
| Race |  |  |  |  |  |  |
| *White* | 64577 (57.40%) | 283831 (56.30%) | 0.024 | 64090 (57.40%) | 64089 (57.40%) | <0.001 |
| *Black or African American* | 24452 (21.70%) | 107513 (21.30%) | 0.011 | 24285 (21.70%) | 24654 (22.10%) | 0.008 |
| *Hispanic or Latino* | 10765 (9.60%) | 57148 (11.30%) | 0.057 | 10729 (9.60%) | 9977 (8.90%) | 0.023 |
| Sex |  |  |  |  |  |  |
| *Female* | 63298 (56.30%) | 239656 (47.50%) | 0.177 | 62745 (56.20%) | 63329 (56.70%) | 0.011 |
| BMI |  |  |  |  |  |  |
| *BMI (25-30 kg/m2)* | 31989 (28.40%) | 186928 (37.10%) | 0.184 | 31926 (28.60%) | 32556 (29.20%) | 0.012 |
| *BMI (>30 kg/m2)* | 80962 (72.00%) | 251479 (49.80%) | 0.466 | 80184 (71.80%) | 80139 (71.80%) | 0.001 |
| Essential (primary) hypertension (I10) | 91615 (81.50%) | 376618 (74.70%) | 0.165 | 90889 (81.40%) | 90554 (81.10%) | 0.008 |
| Hyperlipidemia, unspecified (E78.5) | 75414 (67.10%) | 295004 (58.50%) | 0.178 | 74752 (66.90%) | 73302 (65.60%) | 0.027 |
| Sleep apnea (G47.3) | 56366 (50.10%) | 137321 (27.20%) | 0.484 | 55593 (49.80%) | 54808 (49.10%) | 0.014 |
| Other hyperlipidemia (E78.4) | 35402 (31.50%) | 130147 (25.80%) | 0.126 | 35076 (31.40%) | 33753 (30.20%) | 0.026 |
| Atherosclerotic heart disease of native coronary artery (I25.1) | 25414 (22.60%) | 131289 (26.00%) | 0.08 | 25356 (22.70%) | 24805 (22.20%) | 0.012 |
| Chronic kidney disease (CKD) (N18) | 22977 (20.40%) | 123134 (24.40%) | 0.095 | 22909 (20.50%) | 22725 (20.40%) | 0.004 |
| Acute pancreatitis (K85) | 2262 (2.00%) | 14641 (2.90%) | 0.058 | 2261 (2.00%) | 1811 (1.60%) | 0.03 |
| Malignant neoplasm of thyroid gland (C73) | 1110 (1.00%) | 3465 (0.70%) | 0.033 | 1093 (1.00%) | 854 (0.80%) | 0.023 |
| Other chronic pancreatitis (K86.1) | 849 (0.80%) | 8310 (1.60%) | 0.082 | 849 (0.80%) | 607 (0.50%) | 0.027 |
| Alcohol-induced chronic pancreatitis (K86.0) | 55 (0.00%) | 1403 (0.30%) | 0.057 | 55 (0.00%) | 50 (0.00%) | 0.002 |
| Family history of multiple endocrine neoplasia [MEN] syndrome (Z83.41) | 10 (0.00%) | 22 (0.00%) | 0.006 | 10 (0.00%) | 10 (0.00%) | <0.001 |
| Multiple endocrine neoplasia [MEN] type IIA (E31.22) | 10 (0.00%) | 36 (0.00%) | 0.002 | 10 (0.00%) | 11 (0.00%) | 0.001 |
| Multiple endocrine neoplasia [MEN] type IIB (E31.23) | 0 (0.00%) | 10 (0.00%) | 0.006 | 0 (0.00%) | 10 (0.00%) | 0.013 |
| Sildenafil (136411) | 10631 (9.50%) | 32930 (6.50%) | 0.108 | 10481 (9.40%) | 9806 (8.80%) | 0.021 |
| Tadalafil (358263) | 6581 (5.90%) | 17315 (3.40%) | 0.115 | 6452 (5.80%) | 5685 (5.10%) | 0.03 |
| Amiodarone (703) | 3392 (3.00%) | 21994 (4.40%) | 0.071 | 3387 (3.00%) | 3085 (2.80%) | 0.016 |
| Vardenafil (306674) | 1068 (0.90%) | 4165 (0.80%) | 0.013 | 1057 (0.90%) | 775 (0.70%) | 0.028 |
| Avanafil (1291301) | 165 (0.10%) | 382 (0.10%) | 0.021 | 161 (0.10%) | 118 (0.10%) | 0.011 |
